# Genomic evidence for the parallel regression of melatonin synthesis and signaling pathways in placental mammals

**DOI:** 10.12688/openreseurope.13795.1

**Published:** 2021-07-01

**Authors:** Christopher A. Emerling, Mark S. Springer, John Gatesy, Zachary Jones, Deana Hamilton, David Xia-Zhu, Matt Collin, Frédéric Delsuc

**Affiliations:** 1 Museum of Vertebrate Zoology, University of California, Berkeley, Berkeley, CA, 94720, USA; 2 Institut des Sciences de l’Evolution de Montpellier (ISEM), CNRS, IRD, EPHE, Université de Montpellier, Montpellier, France; 3 Biology Department, Reedley College, Reedley, CA, 93654, USA; 4 Department of Evolution, Ecology, and Organismal Biology, University of California, Riverside, Riverside, CA, 92521, USA; 5 Division of Vertebrate Zoology, American Museum of Natural History, New York, NY, 10024, USA

**Keywords:** Melatonin, Pseudogene, Xenarthra, Pholidota, Dermoptera, Sirenia

## Abstract

**Background**: The study of regressive evolution has yielded a wealth of examples where the underlying genes bear molecular signatures of trait degradation, such as pseudogenization or deletion. Typically, it appears that such disrupted genes are limited to the function of the regressed trait, whereas pleiotropic genes tend to be maintained by natural selection to support their myriad purposes. One such set of genes is involved in the synthesis ( *AANAT*, *ASMT*) and signaling ( *MTNR1A*, *MTNR1B*) of melatonin, a hormone secreted by the vertebrate pineal gland. Melatonin provides a signal of environmental darkness, thereby influencing the circadian and circannual rhythmicity of numerous physiological traits. Therefore, the complete loss of a pineal gland and the underlying melatonin pathway genes seems likely to be maladaptive, unless compensated by extrapineal sources of melatonin.

**Methods**: We examined *AANAT*, *ASMT*, *MTNR1A* and *MTNR1B* in 123 vertebrate species, including pineal-less placental mammals and crocodylians. We searched for inactivating mutations and modelled selective pressures (dN/dS) to test whether the genes remain functionally intact.

**Results**: We report that crocodylians retain intact melatonin genes and express *AANAT* and *ASMT* in their eyes, whereas all four genes have been repeatedly inactivated in the pineal-less xenarthrans, pangolins, sirenians, and whales. Furthermore, colugos have lost these genes, and several lineages of subterranean mammals have partial melatonin pathway dysfunction. These results are supported by the presence of shared inactivating mutations across clades and analyses of selection pressure based on the ratio of non-synonymous to synonymous substitutions (dN/dS), suggesting extended periods of relaxed selection on these genes.

**Conclusions:** The losses of melatonin synthesis and signaling dates to tens of millions of years ago in several lineages of placental mammals, raising questions about the evolutionary resilience of pleiotropic genes, and the causes and consequences of losing melatonin pathways in these species.

## Plain language summary

Evolution is typically thought to occur by making an organism more complex, such as through the addition of new traits. However, evolution can also proceed through the loss or degeneration of characteristics. The reduction of eyes in animals living underground and the loss of limbs in snakes are examples of such regressive evolution. When organisms evolve regressively, the genes underlying such traits often become broken (pseudogenes) or deleted from their genome altogether. However, genes are not typically thought to be lost when they perform many functions (pleiotropy). For instance, four genes are involved in the production ( *AANAT*, *ASMT*) and detection ( *MTNR1A*, *MTNR1B*) of melatonin, a hormone that tells an animal's organ systems that it is dark outside (e.g., during the night). Melatonin genes seem unlikely to become broken or lost given that this hormone is responsible for telling many parts of the body to perform functions specific to nighttime. The loss of such genes would likely have a negative, cascading effect on many body functions; therefore, natural selection would presumably retain functional versions of these genes. Surprisingly, however, certain vertebrates are reported to lack the organ responsible for secreting melatonin (pineal gland). We examined the melatonin genes in these vertebrates to determine if they are broken, missing and/or show evidence of degradation over time. We discovered that while pineal-less crocodylians retain all four melatonin genes, other species reported to lack a pineal gland (anteaters, sloths, armadillos, pangolins, dugong, manatee, whales) have broken and/or missing melatonin genes. Furthermore, colugos (flying lemurs) and some mammals that live underground in complete darkness show evidence of melatonin gene dysfunction. Together, these results indicate that these mammals lost their ability to produce and detect melatonin tens of millions of years ago, raising questions as to how they have adapted to a life without this hormone.

## Introduction

Evidence for the molecular basis of regressive evolution, or vestigialization, has become abundant following increases in the availability of whole genome assemblies ( Albalat & Cañestro, 2016). Such molecular regression typically manifests as the accumulation of unitary pseudogenes and whole gene deletions, although an increasing number of studies are finding that it can also result in or arise from the erosion of gene promoters and enhancers. Among vertebrates, examples include mutations associated with the loss of teeth in favor of beaks and baleen ( Deméré *et al*., 2008; Meredith *et al*., 2014), reduction in visual capabilities as species eschew life aboveground in favor of a subterranean existence ( Emerling & Springer, 2014; Fang *et al*., 2014; Partha *et al*., 2017), and the loss or reduction of limbs while adapting to new locomotory strategies ( Berger *et al*., 2018; Emerling, 2017a; Sackton *et al*., 2019; Vonk *et al*., 2013). A unifying theme is that the loci underlying the loss of these traits typically appear to be restricted to a single or limited function: tooth genes specifically associated with enamel and dentin development become inactivated in toothless vertebrates ( Meredith *et al*., 2014), whereas tooth genes with bone-related or other functions remain intact ( Deméré *et al*., 2008; Springer *et al*., 2016); genes encoding light-sensitive opsins used in bright light conditions are lost as taxa adapt to life underground ( David-Gray *et al*., 2002; Emerling & Springer, 2014), but genes otherwise necessary for eye formation remain intact ( Emerling, 2018; Partha *et al*., 2017); and while a gene encoding a claw-specific keratin in squamates is pseudogenized in snakes ( Emerling, 2017a), multipurpose *Hox* genes associated with limb-development typically remain conserved ( Vonk *et al*., 2013). Such observations should not be surprising given that the pleiotropic nature of many genes necessitates their retention, even if one or more associated traits are lost. In such pleiotropic genes, the loss of regulatory non-coding elements associated with specific functions appears more likely to occur than outright disruption of gene function ( Berger *et al*., 2018; Kvon *et al*., 2016; Partha *et al*., 2017). However, reports of the absence of the pineal gland in several lineages of vertebrates ( Oksche, 1965) challenge the assumption that pleiotropic effects lead to gene conservation.

The pineal gland is an endocrine organ located within the diencephalon of vertebrates, whose primary, and perhaps only, function is to secrete melatonin. Melatonin functions as a potent antioxidant and can also act as a hormone that signals environmental darkness ( Hardeland & Poeggeler, 2003; Zhao *et al*., 2019). In vertebrates, melatonin derives from serotonin, which is converted into *N*-Acetylserotonin by aralkylamine *N*-acetyltransferase (AANAT) and then modified into melatonin by *N*-Acetylserotonin O-methyltransferase (ASMT/HIOMT). Melatonin production in the pineal gland is under the control of the circadian master clock (suprachiasmatic nucleus), following a pattern of high production in darkness and low production in light. It is released into the bloodstream and arrives at target tissues to activate downstream pathways via the G protein-coupled melatonin receptors type 1A (MTNR1A/MT1) and 1B (MTNR1B/MT2) ( Cipolla-Neto *et al*., 2014). These melatonin receptors are expressed widely, including in the suprachiasmatic nucleus, thalamus, cerebral cortex, retina, kidneys, adrenal glands, reproductive organs, arteries, immune cells, liver, pancreas, skin and bone, indicating broad signaling from this hormone ( Cipolla-Neto & Do Amaral, 2018; Sapède & Cau, 2013). Indeed, by providing a signal of darkness, melatonin helps to maintain circadian and circannual rhythms, influencing energy metabolism, seasonal reproduction, migration behavior, blood pressure, immune system functioning, among other processes ( Cipolla-Neto *et al*., 2014; Nishiwaki-Ohkawa & Yoshimura, 2016; Sapède & Cau, 2013).

 Despite such seemingly indispensable effects, and the experience of a light/dark cycle by nearly all vertebrates, a number of vertebrates are reported to lack a pineal gland: sloths, armadillos, and anteaters (Xenarthra), pangolins (Pholidota), certain whales (Cetacea), the dugong ( *Dugong dugon*; Sirenia) and crocodylians ( McFarland *et al*., 1969; Oelschläger *et al*., 2008; Oksche, 1965; Panin *et al*., 2012; Phillips *et al*., 1986; Ralph *et al*., 1985). If accurate, the phenotypic consequences of being pineal-less would likely be widespread, as shown by how pinealectomies can impact clock gene expression ( Coelho *et al*., 2015; De Farias *et al*., 2015), insulin function ( Lima *et al*., 1998), serum leptin levels ( Canpolat *et al*., 2001), dopamine levels ( Khaldy *et al*., 2002), spinal development ( Fjelldal *et al*., 2004), gonadal function ( Baburski *et al*., 2015; Haldar & Ghosh, 1990), and immune function ( Del Gobbo *et al*., 1989). However, despite such anatomical observations, scientists have reported circulating serum melatonin in the nine-banded armadillo ( *Dasypus novemcinctus*), bottlenose dolphin ( *Tursiops truncatus*), American alligator ( *Alligator mississippiensis*), and freshwater crocodile ( *Crocodylus johnstoni*) ( Firth *et al*., 2010; Funasaka *et al*., 2011; Harlow *et al*., 1981; Panin *et al*., 2012; Roth *et al*., 1980), even reporting rhythmic secretions in some species. Furthermore, bottlenose dolphins reportedly show ASMT presence in several tissues ( Panin *et al*., 2012), and exogenous melatonin evidently influences activity patterns and body temperature in the nine-banded armadillo ( Harlow *et al*., 1982). However, a review on melatonin binding sites in the pars tuberalis, suprachiasmatic nucleus, hippocampus and other brain sites found low to no melatonin binding in the nine-banded armadillo, in contrast to 14 other mammals ( Bittman, 1993).

Perhaps reconciling these apparently conflicting data, research has revealed that multiple organs in vertebrates can synthesize melatonin, such as the retina and lens of the eye, thymus and bone marrow ( Acuña-Castroviejo *et al*., 2014; Cogburn *et al*., 1987; Gern *et al*., 1978a; Reppert & Sagar, 1983; Stefulj *et al*., 2001; Underwood *et al*., 1984). Indeed, there is evidence that extrapineal melatonin can enter blood circulation ( Foà & Menaker, 1988; Gern *et al*., 1978b; Gern & Norris, 1979; Kennaway *et al*., 1977; Lynch *et al*., 1975; Owens & Gern, 1985; Reppert & Sagar, 1983; Underwood *et al*., 1984), suggesting that extrapineal organs may ultimately be the source of serum melatonin in pineal-less vertebrates. Furthermore, melatonin synthesis has also been reported in mitochondria ( Suofu *et al*., 2017), providing another potential source of extrapineal melatonin.

Given the ubiquity and myriad effects of this hormone, we hypothesized that melatonin synthesis has been maintained even in pineal-less vertebrates, but may rely on extrapineal sources to perform the same functions. We set out to determine if the genes necessary for melatonin synthesis ( *AANAT*, *ASMT*) and signaling ( *MTNR1A, MTNR1B*) are functionally intact in several clades of apparently pineal-less vertebrates. We found evidence that these melatonin pathway genes have been repeatedly inactivated and under relaxed selection in numerous lineages for tens of millions of years, raising questions of the evolutionary resilience of pleiotropic systems.

## Materials and methods

We obtained *AANAT*, *ASMT*, *MTNR1A* and *MTNR1B* gene sequences for 110 species of placental mammals, including 13 xenarthrans, three pangolins, three sirenians and 25 cetaceans, as well as 13 crocodylians (Underlying data, Supplementary Tables S1–S5 [ Emerling *et al*., 2021]). Gene assembly was accomplished using a combination of whole genome assemblies and mapping short reads of sequenced genomes onto reference sequences. Human and chicken reference mRNA sequences for all four genes were downloaded from GenBank (Underlying data, Supplementary Tables S2–S5 [ Emerling *et al*., 2021]), and the protein-coding sequence of each mRNA was BLASTed (megablast) against human and chicken genome assemblies in NCBI’s (National Center for Biotechnology Information) WGS (Whole Genome Shotgun) database. For each gene, we downloaded a contiguous sequence that included all of the mRNA coding exons and flanking sequence, imported the sequences into Geneious v. 2019.2.3 ( Kearse *et al*., 2012), and aligned each mRNA to its corresponding WGS contig hits using MUSCLE v. 3.5 ( Edgar, 2004). We then used the human and chicken assembly-derived sequences as the references for obtaining orthologs in mammals and crocodylians, respectively.

 For whole genome assemblies, we BLASTed our reference sequences against assemblies in external and imported databases (Underlying data, Supplementary Table S1 [ Emerling *et al*., 2021]) using intermediate sensitivity settings (e.g., discontiguous megablast on NCBI). In the case of some *ASMT* sequences, we queried NCBI’s annotated scaffolds directly (RefSeq). For short reads, we imported the reads into Geneious and mapped them using intermediate sensitivity settings (medium-low sensitivity). For each set of probes, we performed an initial mapping run to expedite sequence capture, followed by a remapping of the captured reads with the “Fine Tuning” option set to iterate up to five times to refine the sequence mapping. When obtaining *AANAT* sequences, the whole reference sequence was used as a probe in BLAST searches and short read mapping. *ASMT* sequences were obtained using a mixture of single exons plus flanking DNA and whole gene reference sequences due to the relatively large size of the gene and small exons. *MTNR1A* and *MTNR1B* both have two coding exons separated by a large intron, so to avoid incompatible homology issues when attempting to align the introns, we typically designed separate probes with flanking sequences for the two coding exons. If we ever failed to recover sequences for a species, we used mRNA sequences and annotated gene predictions, plus newly-assembled sequences, as reference sequences for BLAST and mapping, especially from close relatives. Sequences derived from genome assemblies that contain long stretches of Ns can cause issues with alignments, so we trimmed any such instances to ten Ns. Mapped short reads were examined by eye and trimmed of nonhomologous sequences likely derived from adapters or sequencing errors.

 Upon obtaining the mammalian and crocodylian gene sequences, each sequence was imported into Geneious and aligned to its respective probe (i.e., human or chicken whole gene sequence) using MUSCLE. When working with different clades, alignments for each species in that clade were performed successively to provide anchoring and improve subsequent alignments, followed by manual adjustment (see clades in the underlying data, Supplementary Tables S1–S5 [ Emerling *et al*., 2021]). After creating each clade-specific alignment (Underlying data, Supplementary Dataset S1 [ Emerling *et al*., 2021]), we examined the sequences for frameshift indels, splice donor mutations, and splice acceptor mutations. After excising the introns, the coding sequence was translated to search for start codon mutations, premature stop codons, and canonical stop mutations (Underlying data, Supplementary Tables S2–S5 [ Emerling *et al*., 2021]).

 Following examination of each clade-specific alignment, global alignments for each gene were assembled to perform phylogenetic (RAxML) and selection pressure (PAML) analyses, respectively (Underlying data, Supplementary Datasets S2–S8 [ Emerling *et al*., 2021]). All stop and incomplete codons were replaced with gaps, as were any codons in individual sequences that were difficult to align with confidence. RAxML analyses were performed with v. 8.2.12 ( Stamatakis, 2014) on CIPRES (RAxML-HPC2 on XSEDE) ( Miller *et al*., 2010), using the default parameters (GTR-CAT), and executing 500 bootstrap replications. We performed dN/dS ratio analyses using codeml in PAML ver. 4.8 ( Yang, 2007), implementing branch tests with ω estimation for different branches defined as functional, pseudogenic, mixed/transitional and premutation, following the terminology of Meredith *et al*. (2009). One important distinction, however, is that we applied separate pseudogene category branches for separate pseudogenization events (e.g., for *AANAT*, one for crown Cetacea, one for crown Pholidota, etc.). In addition, for any species that had *AANAT*, *ASMT* and/or both melatonin receptor genes inactivated, separate ω estimates were calculated for each of the other putatively functional genes. For instance, *ASMT* and *MTNR1B* are inactivated in *Fukomys damarensis*, whereas *AANAT* and *MTNR1A* do not appear to be. However, since *ASMT* is inactivated, we estimated ω in *F. damarensis* for *AANAT* and *MTNR1A* in addition to *ASMT* and *MTNR1B*. 

The mammalian tree topology for the PAML analyses (Underlying data, Supplementary Dataset S9 [ Emerling *et al*., 2021]) was largely derived from a single phylogeny ( Emerling *et al*., 2015), with cetacean, carnivoran, xenarthran and talpid relationships obtained from additional sources ( Allio *et al*., 2021; Gibb *et al*., 2016; He *et al*., 2017; McGowen *et al*., 2020). In some cases, relationships for certain mammals were not resolved in the reference trees, but confamilials and/or congeners were present, allowing us to confidently place such taxa in the phylogeny. For example, our primary reference tree does not include *Desmodus rotundus*, but other phyllostomid species are present, so *D. rotundus* was positioned in the phyllostomid portion of the phylogeny. Prior to executing the branch tests, we executed one-ratio branch models with one of three codon frequency models (1/61 each, F1X4, F3X4) and used the Akaike information criterion to select the best-fitting codon frequency model for each gene (Underlying data, Supplementary Table S6 [ Emerling *et al*., 2021]).

 In addition to estimating the dN/dS ratios for branches of interest, we tested whether certain branches had ω values that were statistically distinguishable from the background ω or from the neutral ω value of 1, signifying relaxed selection. In such cases, we had a master model for each gene that included all branch categories (e.g., 24 ratio model *ASMT*, 46 ratio model *MTNR1B*), and nested models in which we changed only one branch of interest to be fixed as the background or 1. We then compared each of the nested models to the master model using likelihood ratio tests to determine which models better fit the data for a branch of interest (Underlying data, Supplementary Tables S7–10 [ Emerling *et al*., 2021]).

 In order to test for evidence of active transcription of the melatonin synthesis genes in a crocodylian, we analyzed RNA sequencing data from 22 sequencing experiments on tissues in a juvenile *Alligator mississippiensis*. We BLASTed (megablast) the protein-coding regions of the *A, mississippiensis AANAT* and *ASMT* genes against short reads derived from mRNA sequencing in NCBI’s Sequence Read Archive (Underlying data, Supplementary Table S11 [ Emerling *et al*., 2021]), imported the sequences into Geneious, and mapped them to the *A. mississippiensis* references using the low sensitivity setting.

 Finally, following the completion of our analyses, a whole genome assembly (WGS) was released for the Steller’s sea cow ( *Hydrodamalis gigas*). The high similarity to other sirenian gene sequences (pairwise comparison with *Dugong dugon*: *AANAT*: 98%; *ASMT*: 97.4%; *MTNR1A*: 96%; *MTNR1B*: 96.6%) allowed us to positively identify these genes and characterize their functionality.

## Results

**Figure 1.**
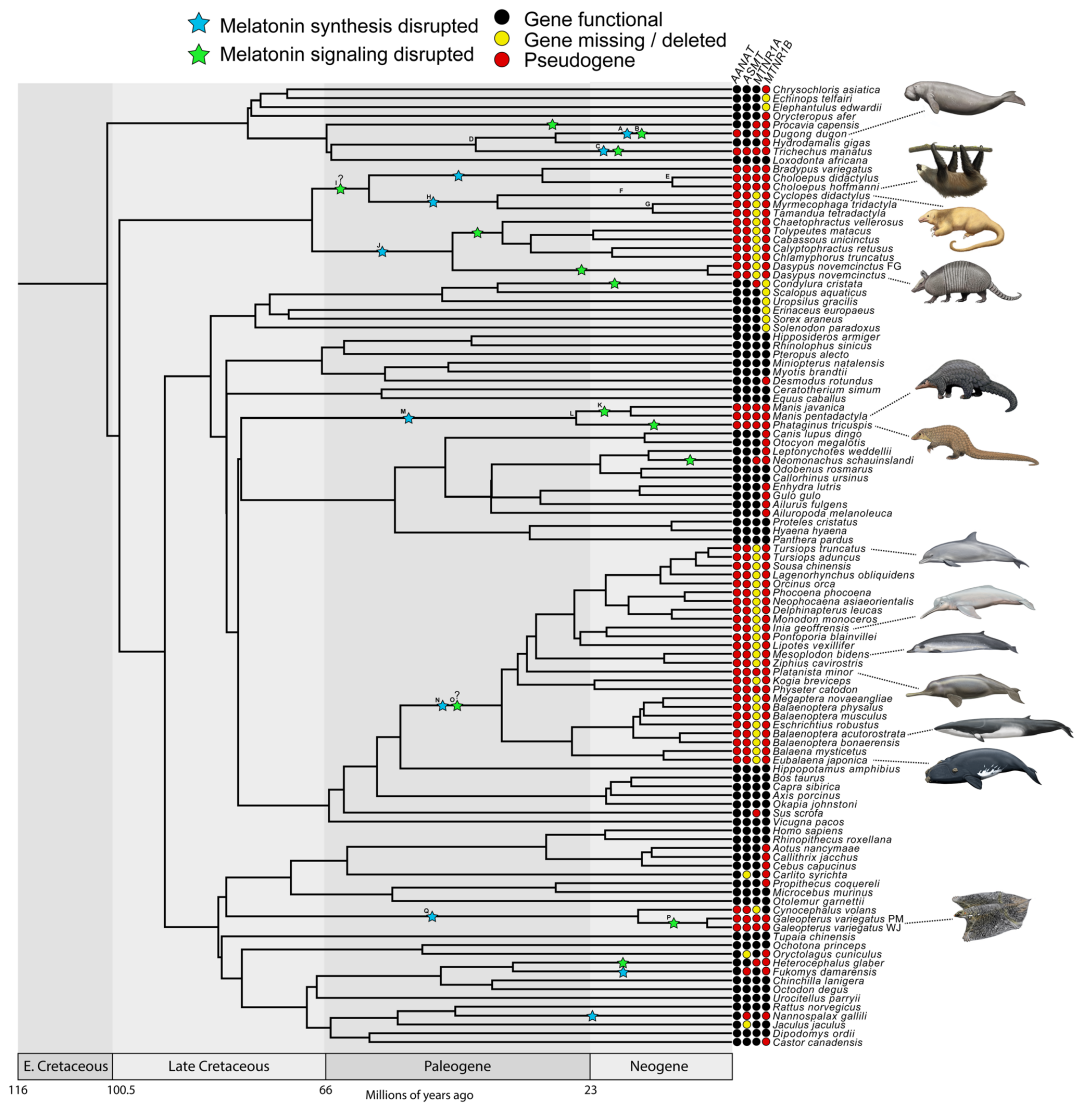
Timetree showing patterns of melatonin gene retention and loss across the placental mammals examined in this study. “Melatonin synthesis disrupted” indicates *AANAT* and/or *ASMT* is inferred to have been inactivated on the associated branch. “Melatonin signaling disrupted” indicates *MTNR1A* and *MTNR1B* are both inferred to have been inactivated on the associated branch, or one gene was lost on an earlier branch and the second was inactivated on the associated branch. Note that the stars are arbitrarily placed in the middle of branches and do not correspond to a precise timing for gene loss. Letters on stars and nodes correspond to letters in Figure 2. References for topology in Materials and Methods. Divergence dates in the figure derived from multiple references ( Gibb *et al*., 2016; Kumar *et al*., 2017; McGowen *et al*., 2020; Meredith *et al*., 2011; Springer *et al*., 2015). Paintings by Carl Buell, copyright John Gatesy.

**Figure 2.**
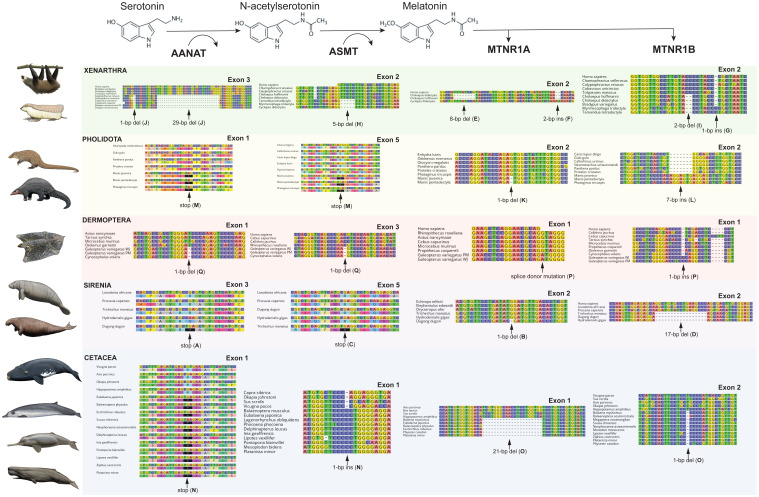
Sampling of inactivating mutations in melatonin pathway genes from five clades of placental mammals. Each column of DNA and protein sequence alignments corresponds to a bolded protein in the melatonin pathways towards the top of the figure. Letters after mutations correspond to letters in the timetree in Figure 1. Paintings by Carl Buell, copyright John Gatesy.

**Figure 3.**
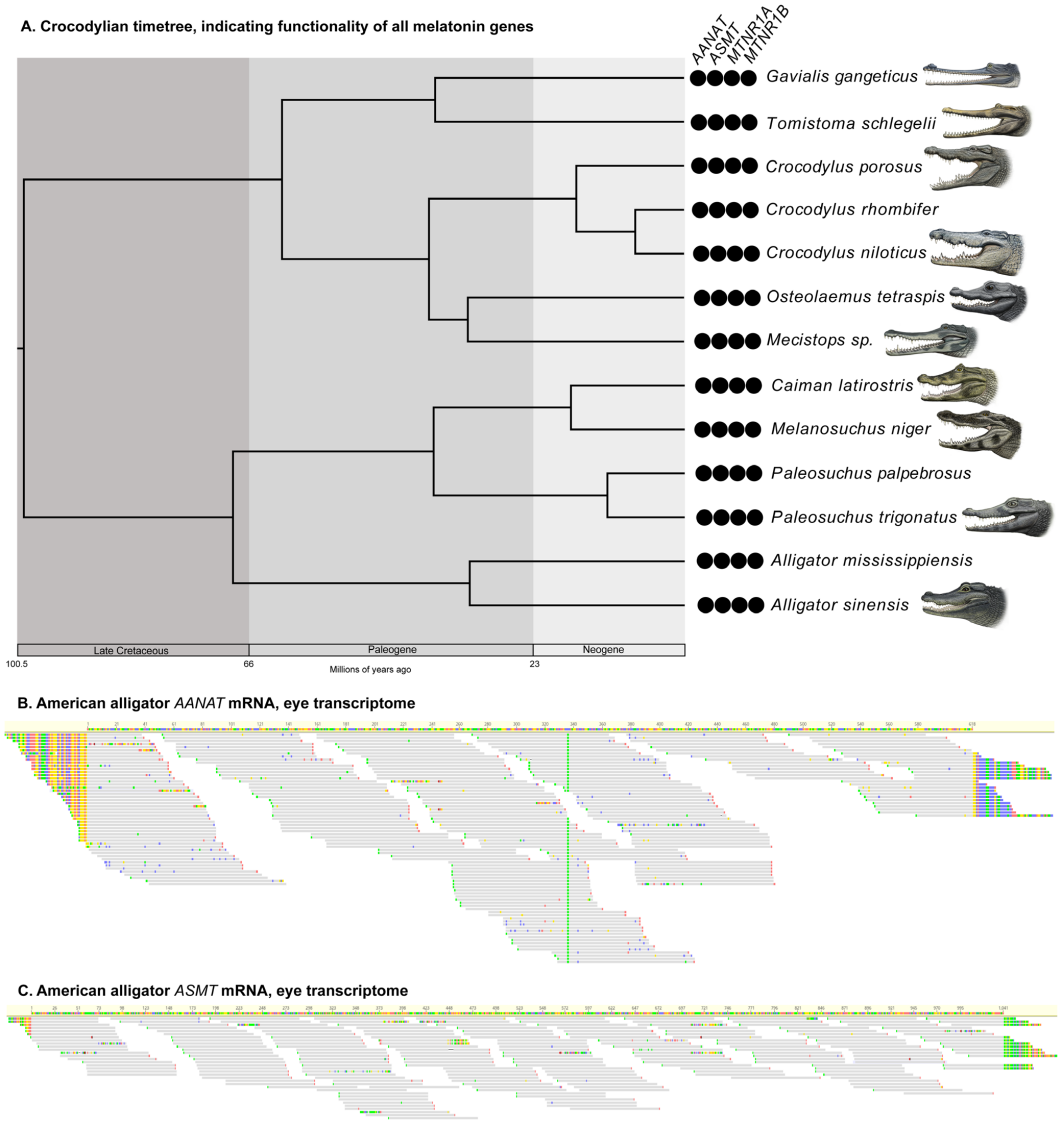
Melatonin genes in crocodylians. **A**. Timetree depicting relationships and divergence times of crocodylians ( Hekkala *et al.*, 2021) examined in this study. The black circles indicate retention of functional orthologs of the genes. Paintings by Carl Buell, copyright John Gatesy. **B**. mRNA short read from an American alligator eye mapped to a reference *AANAT*. **C**. mRNA short reads from an American alligator eye mapped to a reference *ASMT*.

We found evidence that all xenarthrans, all pangolins, all cetaceans and the West Indian manatee ( *Trichechus manatus*) have had both their melatonin synthesis ( *AANAT*, *ASMT*) and melatonin signaling ( *MTNR1A*, *MTNR1B*) genes disrupted via the accumulation of inactivating mutations and whole gene deletions ( Figure 1 & Figure 2; Underlying data, Supplementary Tables S2–S5 [ Emerling *et al*., 2021]). The dugong ( *Dugong dugon*) appears to retain an intact *ASMT*, but otherwise has pseudogenes for the remaining three genes, and the Steller’s sea cow ( *Hydrodamalis gigas*) only has an inactivated *MTNR1B*. By contrast, all 13 crocodylians investigated retain intact melatonin genes ( Figure 3; Underlying data, Supplementary Tables S2–S5 [ Emerling *et al*., 2021]). Furthermore, we found that *AANAT* and *ASMT* are both expressed in the American alligator, particularly within the eye (Underlying data, Supplementary Table S11 [ Emerling *et al*., 2021]). We also found evidence for the complete disruption of both melatonin gene pathways (i.e., synthesis, signaling) in two dermopteran (colugo) species ( *Galeopterus variegatus* Peninsular Malaysia, *G. variegatus* West Java; considered distinct cryptic species by Mason *et al.*, 2016), with a third ( *Cynocephalus volans*) perhaps only retaining a functional *MTNR1B* ( Figure 1 & Figure 2; Underlying data, Supplementary Tables S2–S5 [ Emerling *et al*., 2021]). The melatonin synthesis gene ( *ASMT*) is also inactivated in two subterranean rodents ( *Nannospalax galili*, *Fukomys damarensis*), and both melatonin receptor genes appear to be inactivated in a hyrax ( *Procavia capensis*), a subterranean mole ( *Condylura cristata*), a mole-rat ( *Heterocephalus glaber*), and a seal ( *Neomonachus schauinslandi*) (Underlying data, Supplementary Tables S3–S5 [ Emerling *et al*., 2021]).

The pseudogene status of nearly all of these genes is supported by the positive identity of the genes as orthologs inferred by RAxML analyses (Extended data, Supplementary Figures S1–S3 [ Emerling *et al*., 2021]), the absence of functional paralogs (Extended data, Supplementary Figures S1–S3; Underlying data, Supplementary Tables S2–S5 [ Emerling *et al*., 2021]), presence of shared inactivating mutations across clades ( Figure 2; Underlying data, Supplementary Tables S2–S5 [ Emerling *et al*., 2021]) and dN/dS ratio estimates suggestive of extensive relaxed selection (Extended data, Supplementary Figures S4–S7; Underlying data, Supplementary Tables S7–S10 [ Emerling *et al*., 2021]). Specifics are discussed below.

### Xenarthra

For *AANAT*, sloths (Folivora) share a mutated start codon (ACA), splice donor mutation (GT to GG) in intron 1, 1-bp deletion in exon 2, and two frameshift indels in exon 3. All anteaters (Vermilingua) possess a start codon mutation (GTG or TTG), with six additional inactivating mutations shared between *Myrmecophaga* and *Tamandua*. Armadillos (Cingulata) share a 1-bp deletion in exon 1, and four frameshift indels in exon 3 ( Figure 2). In *ASMT*, there is a putative 1-bp insertion in exon 6 shared by two-toed sloths ( *Choloepus* spp.), two chlamyphorid armadillos ( *Calyptophractus*, *Chlamyphorus*), and a dasypodid armadillo ( *Dasypus novemcinctus* French Guiana). This region is missing in all other xenarthran sequences (i.e., deleted or not assembled), but the alignment is admittedly dubious. Furthermore, exon 5 appears to be absent in all taxa we examined, and the canonical stop codon (i.e., the stop codon common to all other species examined) is mutated in all xenarthrans we examined, although it differs among sloths (TAT), anteaters (TGT or TGG), and armadillos (CGT or TGT). Nonetheless, shared inactivating mutations are present among the anteaters (5-bp deletion, exon 2 [ Figure 2]; two frameshift indels, exon 8), sloths (1-bp deletion, exon 3; 4-bp insertion, exon 7), and armadillos (1-bp deletion, exon 6), respectively. We were unable to obtain *MTNR1A* in most xenarthrans, possibly due to a whole gene deletion, although the silky anteater ( *Cyclopes didactylus*) and sloths retain one or more *MTNR1A* pseudogenes ( Figure 2). Sloths appear to have multiple paralogs, the identities of which are difficult to tease out, with separate exons being found on separate contigs, but the pattern suggests all are probably inactivated. For *MTNR1B*, a 2-bp deletion in exon 2 is shared by sloths and anteaters (Pilosa; Figure 2). Among armadillos, a single 1-bp deletion (exon 1) is shared by dasypodids, and five inactivating mutations are shared among chlamyphorid armadillos (two in exon 1; three in exon 2).

 From dN/dS ratio analyses, we found evidence of shifts in selection pressures consistent with ancient pseudogenization of these genes. Estimates in which key xenarthran branches had statistically elevated ω values were found for *AANAT* (background: 0.36; stem Folivora: 1.57; crown Folivora: 0.71; stem Vermilingua: 2.81; crown Vermilingua: 1.71; stem Cingulata: 7.33; crown Cingulata: 1.34), *ASMT* (background: 0.25; crown Folivora: 0.56; crown Vermilingua: 1.07; crown Cingulata: 0.91; stem Cingulata: 1.06), *MTNR1A* (background: 0.17; crown *Choloepus*: 1.06; stem *Choloepus*: 0.76; *Cyclopes didactylus*: 1.11) and *MTNR1B* (background: 0.32; crown Pilosa: 1.18; crown Chlamyphoridae: 0.79; stem Dasypodidae: 2.36). We also ran a model for *ASMT* in which crown Xenarthra was given a single ω, under the assumption that *it* was inactivated in the stem lineage. This ω was estimated as 0.79, and when compared to a model in which the crown Xenarthra ω was fixed at 1, the former was a better fit for the data than the latter. As such, this result seems to be inconsistent with a stem inactivation of *ASMT*.

### Pholidota

For pangolins, all three species share two premature stop codons in *AANAT* (exons 1 and 2), another in exon 5 of *ASMT*, and four inactivating mutations in *MTNR1B* ( Figure 2). Exon 1 of *MTNR1A* is missing for both *Manis* spp., preventing comparisons with *Phataginus tricuspis*, but the former two share a 1-bp deletion in exon 2 ( Figure 2). Moreover, dN/dS analyses provide further evidence of pseudogenization with elevated ω values for *AANAT* (background: 0.36; crown Pholidota: 0.62), *ASMT* (background: 0.25; Pholidota: 0.72), *MTNR1A* (background: 0.17; crown *Manis*: 1.11; stem *Manis*: 3.35; *Phataginus*: 1.43; stem Pholidota: 0.35), and *MTNR1B* (background: 0.32; crown Pholidota: 1.51; stem Pholidota: 1.14).

### Cetacea

Inactivation of the melatonin genes in cetaceans has already been reported in two recent studies ( Huelsmann *et al*., 2019; Lopes-Marques *et al*., 2019), although we have expanded on these results by increasing the taxon sampling from 12 to 25 species and eight to 13 families ( Figure 1), as well as analyzing the selection patterns of this clade. As in previous studies, we found shared inactivating mutations, including a premature stop codon in exon 1 of *AANAT* (all 25 species), a 1-bp insertion in exon 1 of *ASMT* (21 species, mysticetes + odontocetes), and a 1-bp deletion in exon 2 of *MTNR1B* (17 species, mysticetes + odontocetes) ( Figure 2). For *MTNR1A*, almost all toothed whales (Odontoceti) completely lack the gene in their assemblies, with the exceptions of pseudogenes in *Platanista minor* and *Physeter catodon*. All baleen whales (Mysticeti), *Platanista* and *Physeter* lack exon 2, suggesting that a whole exon deletion occurred on the stem Cetacea branch. Furthermore, an unusual 21-bp deletion in exon 1 shared by odontocetes and mysticetes may represent an in-frame disabling mutation ( Figure 2). dN/dS analyses are consistent with relaxed selection in this clade, with statistically elevated ω values in *AANAT* (background: 0.36; crown Cetacea: 0.99; stem Cetacea: 3.2), *ASMT* (background: 0.25; crown Cetacea: 0.93; stem Cetacea: 0.7), *MTNR1A* (background: 0.17; stem Cetacea: 1.33) and *MTNR1B* (background: 0.32; crown Cetacea: 1.02; stem Cetacea: 0.64).

### Sirenia

For sirenians, shared inactivating mutations are less common, existing only for *MTNR1B* (exon 2: 14-bp deletion, 17-bp deletion [ Figure 2], canonical stop mutation TAC or TGC). Outside of this gene, inactivating mutations are only found in the manatee ( *AANAT*: two; *ASMT*: one; *MTNR1A*: two) and dugong ( *AANAT*: two; *MTNR1A*: two) ( Figure 2). Some of the manatee mutations were previously reported ( Huelsmann *et al*., 2019; Lopes-Marques *et al*., 2019). Here, dN/dS analyses only provide evidence of relaxed selection in *MTNR1A* (background: 0.17; stem Sirenia: 0.48; *Dugong*: 0.53; *Trichechus*: 1.95) and *MTNR1B* (background: 0.32; crown Sirenia: 1.95).

### Dermoptera

Both melatonin synthesis genes have shared inactivating mutations across all three colugos: exon 1 of *AANAT* and exon 3 of ASMT each have shared 1-bp deletions, ( Figure 2). We were unable to map *MTNR1A* for *Cynocephalus volans*, but both *Galeopterus* species have a splice donor mutation (AG to AT) in the intron ( Figure 2). Similarly, we were unable to map exon 2 of *MTNR1B* for *C. volans*, and exon 1 appears intact, but *Galeopterus* spp. share a 1-bp insertion (exon 1; Figure 2) and a premature stop codon (exon 2). Furthermore, dN/dS estimates provide evidence of relaxed selection in *AANAT* (background: 0.36; crown Dermoptera: 0.77), *ASMT* (background: 0.25; crown Dermoptera: 0.6) and *MTNR1A* (background: 0.17; crown *Galeopterus*: 0.81; stem *Galeopterus*: 0.64).

### Other placental mammals

Beyond these taxa, *AANAT* is present as two to three paralogs in multiple non-cetacean cetartiodactyls, with one paralog sometimes being a pseudogene. However, at least one gene is always intact in all non-cetacean cetartiodactyls that were examined. *ASMT* is a pseudogene in two subterranean rodents ( *Fukomys damarensis*, Bathyergidae; *Nannospalax galili*, Spalacidae), *MTNR1A* is inactivated in a hyrax ( *Procavia capensis*), monk seal ( *Neomonachus schauinslandi*), pig ( *Sus scrofa*), talpid mole ( *Condylura cristata*), and the naked-mole rat ( *Heterocephalus glaber*), and *MTNR1B* is a pseudogene or likely deleted (i.e., negative BLAST results) in a host of other species, including six afrotherians, eight carnivorans, one bat, all six examined eulipotyphlans, one lagomorph, five primates, and five rodents ( Figure 1). The only example of a shared inactivating mutation we found in these other species is a 1-bp insertion in exon 2 of *MTNR1B* for two platyrrhine monkeys ( *Callithrix jacchus*, *Cebus capucinus*). Here also, dN/dS ratio analyses suggest that many of these pseudogenes are indeed under relaxed selection based on statistically elevated ω values.

## Discussion

### Melatonin genes inactivated in many mammals, but functional in crocodylians

Here we reported evidence that xenarthrans, pangolins, cetaceans and some sirenians have lost the capability to synthesize and bind melatonin via the traditional pathway found in vertebrates, coinciding with the ostensible absence of a pineal gland in these taxa. This builds upon recent studies on cetaceans and the West Indian manatee ( Huelsmann *et al*., 2019; Lopes-Marques *et al*., 2019), demonstrating the surprising extent of the degradation of these genes. We hypothesized that despite the pineal gland’s apparent absence in xenarthrans, pangolins, sirenians, cetaceans and crocodylians, the genes underlying the production and signaling of melatonin would remain intact, given the widespread effects of melatonin in vertebrates and evidence of circulating melatonin in these taxa ( Firth *et al*., 2010; Funasaka *et al*., 2011; Harlow *et al*., 1981; Panin *et al*., 2012; Roth *et al*., 1980). Indeed, this appears to be the case for crocodylians, in which all 13 species we investigated, representing all major lineages, possess intact orthologs of *AANAT*, *ASMT*, *MTNR1A* and *MTNR1B*. Given the presence of serum melatonin in crocodylians ( Firth *et al*., 2010; Roth *et al*., 1980), this suggests that either the pineal gland is intact but difficult to isolate and/or extra-pineal sources of melatonin exist. In support of the latter hypothesis, we found evidence of *AANAT* and *ASMT* expression in the eyes of the American alligator ( *Alligator mississippiensis*). Furthermore, a previous study found that the gene encoding a pineal opsin pigment is a pseudogene in crocodylians ( Emerling, 2017b), potentially revealing a shift in the source of melatonin from the pineal gland to the eye, following the regression of the former.

By contrast, most major melatonin pathway genes are pseudogenized in pineal-less mammals. Researchers have varied in their reports on the presence versus absence of pineal glands in cetaceans ( Behrmann, 1990; Holzmann, 1991; Lyamin *et al*., 2008; McFarland *et al*., 1969; Oelschläger *et al*., 2008; Panin *et al*., 2012) and work on sirenians appears to be inconsistent, with some studies suggestive of a minute pineal gland and others of its complete absence ( Chapman, 1875; Murie, 1872; Ralph *et al*., 1985). A recent anatomical study failed to find a pineal gland in the white-bellied pangolin ( *Phataginus tricuspis*), although the absence was attributed to an error in preparing the brain for study ( Imam *et al*., 2019). Despite these inconsistencies, a regressed, if not completely absent, pineal gland largely predicts the disappearance of the canonical melatonin pathways, at least in mammals. Furthermore, we found evidence of melatonin synthesis inactivation in colugos, suggesting that these species may also possess a regressed pineal gland, or may lack it entirely. Although one study found a diencephalon in colugos comparable in relative size to gliding rodents and bats ( Pirlot & Kamiya, 1982), a review of pinealocytes in mammals noted the lack of research specifically on the pineal in Dermoptera ( Bhatnagar, 1992).

While non-pineal melatonin may reconcile the patterns of circulating melatonin and the putative absence of a pineal gland in crocodylians, it cannot explain this same phenomenon in the nine-banded armadillo ( Harlow *et al*., 1982; Harlow *et al*., 1981) and bottlenose dolphins ( Funasaka *et al*., 2011; Panin *et al*., 2012). Possible sources for the serum melatonin detected in these taxa may derive from an unknown alternative pathway ( Tan *et al*., 2016), dietary sources ( Hattori *et al*., 1995; Peuhkuri *et al*., 2012; Reiter *et al*., 2005), or the organism’s microbiome ( Hardeland & Poeggeler, 2003). However, the functional significance of serum melatonin may be obviated by the absence of melatonin receptors in these species, presumably preventing any contribution to circadian signaling in the body’s tissues.

### Ancient loss of melatonin genes in placental mammals

Our results strongly suggest that melatonin synthesis and signaling has been abolished within multiple placental mammal lineages for extensive periods of evolutionary time. The most ancient of these may be in Xenarthra, given our evidence of *ASMT* and possibly *MTNR1A* inactivation on the stem branch. Crown Xenarthra arose roughly 68 million years ago (mya) ( Gibb *et al*., 2016), potentially meaning that the loss of melatonin synthesis and possibly some signaling took place near the K/Pg boundary, when non-avian dinosaurs went extinct and placental mammals began to radiate. However, given some ambiguity in the evidence for stem inactivation of *ASMT* (see results), and the absence of sequences of *MTNR1A* for most species of xenarthrans, convergent loss remains a strong possibility. Despite this, additional shared mutations in *AANAT* and *ASMT* suggest that the components for melatonin synthesis were disrupted prior to the origin of armadillos (45 mya), sloths (31 mya), and anteaters (38 mya). In addition, *MTNR1B* was likely pseudogenized prior to the sloth / anteater split (59 mya), and the origin of stem chlamyphorid (37 mya) armadillos. In pangolins, shared mutations in *AANAT*, *ASMT*, and *MTNR1B* suggest complete loss of melatonin synthesis and at least some melatonin signaling prior to the origin of this clade 25 mya ( Meredith *et al*., 2011). For colugos, *AANAT* and *ASMT* were likely inactivated prior to the origin of crown Dermoptera, indicating the absence of melatonin synthesis for at least 15 million years ( Mason *et al*., 2016). There are contrasting patterns among the aquatic taxa, with cetaceans likely having lost all four genes prior to their origin 37 mya ( McGowen *et al*., 2020), whereas for sirenians, we only have positive evidence of *MTNR1B* inactivation in the stem lineage at least 42 mya ( Springer *et al*., 2015). Subsequent parallel pseudogenization events occurred in other melatonin-related genes for the manatee and dugong, but not Steller’s sea cow.

### Causes and consequences of losing the melatonin pathway

The potential significance of the loss of both melatonin synthesis and signaling in multiple clades of mammals should not be understated. To reiterate, melatonin is a ubiquitous biogenic compound found in Eubacteria, unicellular eukaryotes, plants, fungi and animals ( Hardeland & Poeggeler, 2003). While it is unclear if melatonin synthesis has a single origin or evolved independently in several lineages ( Tan *et al*., 2014; Zhao *et al*., 2019), its widespread taxonomic occurrence suggests that it has ancient origins and that natural selection has favored the maintenance of synthesis pathways for perhaps billions of years. Furthermore, after a hypothesized co-option of this potent antioxidant to signal environmental darkness ( Zhao *et al*., 2019), in order to help modulate circadian and circannual physiological processes, melatonin synthesis and signaling would seemingly become indispensable for most vertebrates. As such, it is a challenge to clarify the causes and consequences of losing melatonin pathway genes.

Convergent evolution often results from similar selection pressures, which may explain why both cetaceans and some sirenians have lost these genes. Perhaps the unique demands of a fully aquatic lifestyle, such as the need to frequently surface for respiration, needed to be uncoupled from a rhythmic signal of darkness. By contrast, the semi-aquatic sea otter ( *Enhydra lutris*) and pinnipeds have retained their melatonin synthesis genes, although *MTNR1B* is pseudogenized in *E. lutris* and two seals (Phocidae), with one of the phocids also showing evidence of *MTNR1A* inactivation. Perhaps this underlies their intermediate aquatic phenotype, although eight of the 13 carnivorans also present an *MTNR1B* pseudogene. Another example of strong convergent evolution can be seen in xenarthran anteaters and pangolins, both of which have radically modified their feeding apparatus to ingest ants and termites (myrmecophagy). However, other myrmecophagous taxa we examined, including the aardvark ( *Orycteropus afer*), aardwolf ( *Proteles cristatus*), and bat-eared fox ( *Otocyon megalotis*) at most only have *MTNR1B* inactivated.

Regardless of their specific phenotypes, all of these taxa experience fluctuations in light and darkness, so it is unclear as to why loss of such a hormone would be beneficial. By contrast, it seems more logical for melatonin synthesis to be lost while adapting to an environment of complete darkness, in which rhythmic secretions entrained on light patterns may no longer be possible. Multiple lineages of subterranean mammals fit this description, and indeed, we found evidence of *ASMT* pseudogenes in the subterranean rodents *Nannospalax galili* (Spalacidae) and *Fukomys damarensis* (Bathyergidae), and inactivation of both receptors in the naked mole-rat ( *Heterocephalus glaber*; Bathyergidae). The latter result had been previously reported in a single individual ( Kim *et al*., 2011), but we confirmed that both genes share the same disabling mutations in a second individual *H. glaber* and are likely under relaxed selection. The fossorial star-nosed mole, *Condylura cristata* (Talpidae), also appears to have dispensed of both melatonin receptors, with *MTNR1A* a pseudogene and *MTNR1B* being completely absent. In addition, dN/dS estimates suggest selection on *ASMT* is relaxed in *H. glaber*, a species which also has an atrophied pineal gland ( Quay, 1981), and *MTNR1B* is inactivated in *N. galili*, *F. damarensis* and a golden mole ( *Chrysochloris asiatica*; Chrysochloridae). This may be relevant to the ancient loss of melatonin synthesis and signaling in xenarthrans, given that comparative anatomy and an analysis of genes critical for vision in bright light appear to point to an early subterranean history for this clade ( Emerling & Springer, 2015). Perhaps an extended history underground limited the utility of melatonin synthesis and signaling, and upon emerging from this committed existence in the darkness, their descendants inherited this unusual phenotype. However, a pineal gland that can synthesize melatonin has been reported in at least one spalacid ( Balemans *et al*., 1980), pineal glands are reported to be present in talpids and chrysochlorids ( Legait *et al*., 1976; Pevet, 1974; Pevet & Kuyper, 1978) and the melatonin synthesis genes remain intact for *C. asiatica* and *C. cristata*.

 One potentially unifying concept for the pattern of pineal gland / melatonin synthesis loss may be related to thermoregulation. Ralph (1975) hypothesized that the size of the pineal gland in vertebrates may be correlated to thermoregulation, tentatively linking pineal gland size to latitude, activity pattern and relative homeothermy. He observed that some of the largest pineal glands belong to species that inhabit higher latitudes, while pointing out that some vertebrates with the smallest or absent pineal glands tend to be restricted to the tropics. Though the comparisons were limited, a better-substantiated pattern was noted with the related parietal eye of squamates. The parietal eye is an organ that is developmentally related to and anatomically linked with the pineal gland, which appears to largely provide information for thermoregulation in ectothermic squamates, possibly through melatonin regulation ( Firth & Kennaway, 1980; Hutchison & Kosh, 1974; Phillips & Harlow, 1981; Stebbins & Eakin, 1958). When comparing the presence or absence of the parietal eye, researchers noted a trend of parietal eye loss in squamates that live near the equator ( Gundy *et al*., 1975). Given that the parietal eye provides information about temperature, which largely correlates with the amount of sunlight, and the pineal gland secretes melatonin in darkness, the latitudinal hypothesis may have some validity. At lower latitudes, there is less seasonality; therefore, being able to detect changes in circadian and circannual dark and light cues is plausibly of less adaptive benefit in these regions. Notably, xenarthrans, pangolins, sirenians and colugos live almost exclusively in the tropics; furthermore, aquatic and subterranean habitats provide a buffering effect from temperature fluctuation. These characteristics encompass all taxa we record as lacking melatonin synthesis capabilities, making it a potentially attractive hypothesis.

Significantly, the patterns of melatonin pathway degradation have strong overlap with placental mammals that have lost the capacity for non-shivering thermogenesis (NST). Specifically, xenarthrans, pangolins, cetaceans and sirenians all have inactivated *UCP1*, a gene that facilitates NST ( Gaudry *et al*., 2017). Furthermore, hyraxes (Hyracoidea) and pigs (Suidae) have a *UCP1* pseudogene, the rock hyrax ( *Procavia capensis*) has both melatonin receptor genes inactivated, and the wild boar ( *Sus scrofa*) has an *MTNR1A* pseudogene. Notably, melatonin induces the production of brown adipose tissue ( Heldmaier *et al*., 1981; Heldmaier & Hoffmann, 1974), a major location of NST. Together, these data suggest that the loss of melatonin synthesis may be coupled with the loss of this thermoregulatory tool in these clades, further underscoring a potential link between melatonin pathway loss and changes in thermoregulatory requirements.

## Conclusion

In this study we provided evidence that, in contrast to crocodylians, numerous placental mammals reported to lack a pineal gland also lack the genes necessary for the canonical vertebrate melatonin synthesis and signaling pathways. However, this result seems to raise more questions than answers. Given the pleiotropic nature of melatonin synthesis and signaling genes, which selection pressure(s) could have led to the loss of this seemingly crucial signaling molecule? What are the physiological consequences of this loss? Are there possibly compensatory alternative mechanisms for producing and sensing melatonin? For those species that present serum melatonin, how are they doing so? Does this melatonin function merely as an antioxidant, or does it aid in circadian and circannual signaling via different pathways? Further studies on comparative anatomy, physiology and gene expression in pineal-less taxa and others should shed further light on these challenging questions.

## Data availability

### Underlying data

Zenodo: Genomic evidence for the parallel regression of melatonin synthesis and signaling pathways in placental mammals. http://doi.org/10.5281/zenodo.4894212 ( Emerling *et al*., 2021)

This project contains the following underlying data:

- Supplementary_Dataset_S1_all_ali_fasta.txt: Genomic alignments in fasta format used to determine the pseudogene/functional status of the different genes in different taxonomic groups.
- Supplementary_Dataset_S2_AANAT_RAxML_ali.phy: Alignment of AANAT in phylip format used in maximum likelihood phylogenetic reconstruction with RAxML.
- Supplementary_Dataset_S3_ASMT_RAxML_ali.phy: Alignment of ASMT in phylip format used in maximum likelihood phylogenetic reconstruction with RAxML.
- Supplementary_Dataset_S4_MTNR1A_MTNR1B_RAxML_ali.phy: Alignment of MTNR1A and MTNR1B in phylip format used in maximum likelihood phylogenetic reconstruction with RAxML.
- Supplementary_Dataset_S5_AANAT_PAML_alig.fasta: Codon alignment of AANAT in fasta format used in selection pressure analyses with PAML.
- Supplementary_Dataset_S6_ASMT_PAML_ali.fasta: Codon alignment of ASMT in fasta format used in selection pressure analyses with PAML.
- Supplementary_Dataset_S7_MTNR1A_PAML_ali.fasta: Codon alignment of MTNR1A in fasta format used in selection pressure analyses with PAML.
- Supplementary_Dataset_S8_MTNR1B_PAML_ali.fasta: Codon alignment of MTNR1B in fasta format used in selection pressure analyses with PAML.
- Supplementary_Dataset_S9_PAML_topology.tre: Tree topology in newick format used in selection pressure analyses with PAML.
- Supplementary_Table_S1.xlsx: List of species examined in this study and the sources of the genes.
- Supplementary_Table_S2.xlsx: Accession numbers and functionality of AANAT in species examined.
- Supplementary_Table_S3.xlsx: Accession numbers and functionality of ASMT in species examined.
- Supplementary_Table_S4.xlsx: Accession numbers and functionality of MTNR1A in species examined.
- Supplementary_Table_S5.xlsx: Accession numbers and functionality of MTNR1B in species examined.
- Supplementary_Table_S6.xlsx: Codon frequency model selection. These are the results from one ratio dN/dS analyses using different codon frequency models.
- Supplementary_Table_S7.xlsx: Results of AANAT PAML dN/dS analyses.
- Supplementary_Table_S8.xlsx: Results of ASMT PAML dN/dS analyses.
- Supplementary_Table_S9.xlsx: Results of MTNR1A PAML dN/dS analyses.
- Supplementary_Table_S10.xlsx: Results of MTNR1B PAML dN/dS analyses.
- Supplementary_Table_S11.xlsx: Results of BLASTing and mapping short reads from *Alligator mississippiensis* RNA sequencing experiments.

### Extended data

Zenodo: Genomic evidence for the parallel regression of the melatonin synthesis and signaling pathways in placental mammals. http://doi.org/10.5281/zenodo.4894212 ( Emerling *et al*., 2021)

This project contains the following extended data:

- Supplementary_Figure_S1.pdf: RAxML AANAT gene tree.
- Supplementary_Figure_S2.pdf: RAxML ASMT gene tree.
- Supplementary_Figure_S3.pdf: RAxML MTNR1A+MTNR1B tree.
- Supplementary_Figure_S4.pdf: PAML AANAT results, model 1.
- Supplementary_Figure_S5.pdf: PAML ASMT results, model 2.
- Supplementary_Figure_S6.pdf: PAML MTNR1A results, model 1.
- Supplementary_Figure_S7.pdf: PAML MTNR1B results, model 1.

Data are available under the terms of the Creative Commons Attribution 4.0 International license (CC-BY 4.0).
