# Genomic evidence for the parallel regression of melatonin synthesis and signaling pathways in placental mammals

**DOI:** 10.12688/openreseurope.13795.2

**Published:** 2021-12-13

**Authors:** Christopher A. Emerling, Mark S. Springer, John Gatesy, Zachary Jones, Deana Hamilton, David Xia-Zhu, Matt Collin, Frédéric Delsuc

**Affiliations:** 1 Museum of Vertebrate Zoology, University of California, Berkeley, Berkeley, CA, 94720, USA; 2 Institut des Sciences de l’Evolution de Montpellier (ISEM), CNRS, IRD, EPHE, Université de Montpellier, Montpellier, France; 3 Biology Department, Reedley College, Reedley, CA, 93654, USA; 4 Department of Evolution, Ecology, and Organismal Biology, University of California, Riverside, Riverside, CA, 92521, USA; 5 Division of Vertebrate Zoology, American Museum of Natural History, New York, NY, 10024, USA

**Keywords:** Melatonin, Pseudogene, Xenarthra, Pholidota, Dermoptera, Sirenia

## Abstract

**Background**: The study of regressive evolution has yielded a wealth of examples where the underlying genes bear molecular signatures of trait degradation, such as pseudogenization or deletion. Typically, it appears that such disrupted genes are limited to the function of the regressed trait, whereas pleiotropic genes tend to be maintained by natural selection to support their myriad purposes. One such set of pleiotropic genes is involved in the synthesis ( *AANAT*, *ASMT*) and signaling ( *MTNR1A*, *MTNR1B*) of melatonin, a hormone secreted by the vertebrate pineal gland. Melatonin provides a signal of environmental darkness, thereby influencing the circadian and circannual rhythmicity of numerous physiological traits. Therefore, the complete loss of a pineal gland and the underlying melatonin pathway genes seems likely to be maladaptive, unless compensated by extrapineal sources of melatonin.

**Methods**: We examined *AANAT*, *ASMT*, *MTNR1A* and *MTNR1B* in 123 vertebrate species, including pineal-less placental mammals and crocodylians. We searched for inactivating mutations and modelled selective pressures (dN/dS) to test whether the genes remain functionally intact.

**Results**: We report that crocodylians retain intact melatonin genes and express *AANAT* and *ASMT* in their eyes, whereas all four genes have been repeatedly inactivated in the pineal-less xenarthrans, pangolins, sirenians, and whales. Furthermore, colugos have lost these genes, and several lineages of subterranean mammals have partial melatonin pathway dysfunction. These results are supported by the presence of shared inactivating mutations across clades and analyses of selection pressure based on the ratio of non-synonymous to synonymous substitutions (dN/dS), suggesting extended periods of relaxed selection on these genes.

**Conclusions:** The losses of melatonin synthesis and signaling date to tens of millions of years ago in several lineages of placental mammals, raising questions about the evolutionary resilience of pleiotropic genes, and the causes and consequences of losing melatonin pathways in these species.

## Plain language summary

Evolution is typically thought to occur by making an organism more complex, such as through the addition of new traits. However, evolution can also proceed through the loss or degeneration of characteristics. The reduction of eyes in animals living underground and the loss of limbs in snakes are examples of such regressive evolution. When organisms evolve regressively, the genes underlying such traits often become broken (pseudogenes) or deleted from their genome altogether. However, genes are not typically thought to be lost when they perform many functions (pleiotropy). For instance, four genes are involved in the production ( *AANAT*, *ASMT*) and detection ( *MTNR1A*, *MTNR1B*) of melatonin, a hormone that signals an animal's organ systems that it is dark outside (e.g., during the night). Melatonin genes seem unlikely to become broken or lost given that this hormone is responsible for signalling many parts of the body to perform functions specific to nighttime. The loss of such genes would likely have a negative, cascading effect on many body functions; therefore, natural selection would presumably retain functional versions of these genes. Surprisingly, however, certain vertebrates are reported to lack the organ responsible for secreting melatonin (pineal gland). We examined the melatonin genes in these vertebrates to determine if they are broken, missing and/or show evidence of degradation over time. We discovered that while pineal-less crocodylians retain all four melatonin genes, other species reported to lack a pineal gland (anteaters, sloths, armadillos, pangolins, dugong, manatee, whales) have broken and/or missing melatonin genes. Furthermore, colugos (flying lemurs) and some mammals that live underground in complete darkness show evidence of melatonin gene dysfunction. Together, these results indicate that these mammals lost their ability to produce and detect melatonin tens of millions of years ago, raising questions as to how they have adapted to a life without this hormone.

## Introduction

Evidence for the molecular basis of regressive evolution, or vestigialization, has become abundant following increases in the availability of whole genome assemblies ( Albalat & Cañestro, 2016). Such molecular regression typically manifests as the accumulation of unitary pseudogenes and whole gene deletions, although an increasing number of studies are finding that it can also result in or arise from the erosion of gene promoters and enhancers. Among vertebrates, examples include mutations associated with the loss of teeth in exchange for beaks and baleen ( Deméré *et al*., 2008; Meredith *et al*., 2014), reduction in visual capabilities as species eschew life aboveground in favor of a subterranean existence ( Emerling & Springer, 2014; Fang *et al*., 2014; Partha *et al*., 2017), and the loss or reduction of limbs while adapting to new locomotory strategies ( Berger *et al*., 2018; Emerling, 2017a; Sackton *et al*., 2019; Vonk *et al*., 2013). A unifying theme is that the loci underlying the loss of these traits typically appear to be restricted to a single or limited function: tooth genes specifically associated with enamel and dentin development become inactivated in toothless vertebrates ( Meredith *et al*., 2014), whereas tooth genes with bone-related or other functions remain intact ( Deméré *et al*., 2008; Springer *et al*., 2016); genes encoding light-sensitive opsins used in bright light conditions are lost as taxa adapt to life underground ( David-Gray *et al*., 2002; Emerling & Springer, 2014), but genes otherwise necessary for eye formation remain intact ( Emerling, 2018; Partha *et al*., 2017); and while a gene encoding a claw-specific keratin in squamates is pseudogenized in snakes ( Emerling, 2017a), multipurpose *Hox* genes associated with limb-development typically remain conserved ( Vonk *et al*., 2013). Such observations should not be surprising given that the pleiotropic nature of many genes necessitates their retention, even if one or more associated traits are lost. In such pleiotropic genes, the loss of regulatory non-coding elements associated with specific functions appears more likely to occur than outright disruption of gene function ( Berger *et al*., 2018; Kvon *et al*., 2016; Partha *et al*., 2017). However, reports of the absence of the pineal gland in several lineages of vertebrates ( Oksche, 1965) challenge the assumption that pleiotropic effects lead to gene conservation.

The pineal gland is an endocrine organ located within the diencephalon of vertebrates, whose primary, and perhaps only, function is to secrete melatonin. Melatonin functions as a potent antioxidant and can also act as a hormone that signals environmental darkness ( Hardeland & Poeggeler, 2003; Zhao *et al*., 2019). In vertebrates, melatonin derives from serotonin, which is converted into *N*-Acetylserotonin by aralkylamine *N*-acetyltransferase (AANAT) and then modified into melatonin by *N*-Acetylserotonin O-methyltransferase (ASMT/HIOMT). Melatonin synthesis in the pineal gland is under the control of the circadian master clock (suprachiasmatic nucleus), following a pattern of high production in darkness and low production in light. It is released into the bloodstream and arrives at target tissues to activate downstream pathways via the G protein-coupled melatonin receptors type 1A (MTNR1A/MT1) and 1B (MTNR1B/MT2) ( Cipolla-Neto *et al*., 2014). These melatonin receptors are expressed widely, including in the suprachiasmatic nucleus, thalamus, cerebral cortex, retina, kidneys, adrenal glands, reproductive organs, arteries, immune cells, liver, pancreas, skin and bone, indicating broad signaling from this hormone ( Cipolla-Neto & Do Amaral, 2018; Sapède & Cau, 2013). Indeed, by providing a signal of darkness, melatonin helps to maintain circadian and circannual rhythms, influencing energy metabolism, seasonal reproduction, migration behavior, blood pressure, immune system functioning, among other processes ( Cipolla-Neto *et al*., 2014; Nishiwaki-Ohkawa & Yoshimura, 2016; Sapède & Cau, 2013).

 Despite such seemingly indispensable effects, and the experience of a light/dark cycle by nearly all vertebrates, a number of vertebrates are reported to lack a pineal gland: sloths, armadillos, and anteaters (Xenarthra), pangolins (Pholidota), certain whales (Cetacea), the dugong ( *Dugong dugon*; Sirenia) and crocodylians ( McFarland *et al*., 1969; Oelschläger *et al*., 2008; Oksche, 1965; Panin *et al*., 2012; Phillips *et al*., 1986; Ralph *et al*., 1985). If accurate, the phenotypic consequences of being pineal-less would likely be widespread, as shown by how pinealectomies can impact clock gene expression ( Coelho *et al*., 2015; De Farias *et al*., 2015), insulin function ( Lima *et al*., 1998), serum leptin levels ( Canpolat *et al*., 2001), dopamine levels ( Khaldy *et al*., 2002), spinal development ( Fjelldal *et al*., 2004), gonadal function ( Baburski *et al*., 2015; Haldar & Ghosh, 1990), and immune function ( Del Gobbo *et al*., 1989). However, despite such anatomical observations, scientists have reported circulating serum melatonin in the nine-banded armadillo ( *Dasypus novemcinctus*), bottlenose dolphin ( *Tursiops truncatus*), American alligator ( *Alligator mississippiensis*), and freshwater crocodile ( *Crocodylus johnstoni*) ( Firth *et al*., 2010; Funasaka *et al*., 2011; Harlow *et al*., 1981; Panin *et al*., 2012; Roth *et al*., 1980), even reporting rhythmic secretions in some species. Furthermore, bottlenose dolphins reportedly show ASMT presence in several tissues ( Panin *et al*., 2012), and exogenous melatonin evidently influences activity patterns and body temperature in the nine-banded armadillo ( Harlow *et al*., 1982). However, a review on melatonin binding sites in the pars tuberalis, suprachiasmatic nucleus, hippocampus and other brain sites found low to no melatonin binding in the nine-banded armadillo, in contrast to 14 other mammals ( Bittman, 1993).

Perhaps reconciling these apparently conflicting data, research has revealed that multiple organs in vertebrates can synthesize melatonin, such as the retina and lens of the eye, thymus and bone marrow ( Acuña-Castroviejo *et al*., 2014; Cogburn *et al*., 1987; Gern *et al*., 1978a; Reppert & Sagar, 1983; Stefulj *et al*., 2001; Underwood *et al*., 1984). Indeed, there is evidence that extra-pineal melatonin can enter blood circulation ( Foà & Menaker, 1988; Gern *et al*., 1978b; Gern & Norris, 1979; Kennaway *et al*., 1977; Lynch *et al*., 1975; Owens & Gern, 1985; Reppert & Sagar, 1983; Underwood *et al*., 1984), suggesting that extra-pineal organs may ultimately be the source of serum melatonin in pineal-less vertebrates. Furthermore, melatonin synthesis has also been reported in mitochondria ( Suofu *et al*., 2017), providing another potential source of extra-pineal melatonin.

Given the ubiquity and myriad effects of this hormone, we hypothesized that melatonin synthesis has been maintained even in pineal-less vertebrates, but may rely on extra-pineal sources to perform the same functions. We set out to determine if the genes necessary for melatonin synthesis ( *AANAT*, *ASMT*) and signaling ( *MTNR1A, MTNR1B*) are functionally intact in several clades of apparently pineal-less vertebrates. We found evidence that in contrast to crocodylians, which maintain intact melatonin pathway genes, numerous placental mammal lineages show evidence of melatonin synthesis and/or signaling disruption. We inferred that several of these events occurred tens of millions of years ago, raising questions about the evolutionary resilience of pleiotropic systems.

## Materials and methods

### Gene dataset assembly

We obtained *AANAT*, *ASMT*, *MTNR1A* and *MTNR1B* gene sequences for 110 species of placental mammals, including 13 xenarthrans, three pangolins, three sirenians and 25 cetaceans, as well as 13 crocodylians (Underlying data, Supplementary Tables S1–S5 [ Emerling *et al*., 2021]). Gene assembly was accomplished using a combination of whole genome assemblies and mapping short reads of sequenced genomes onto reference sequences using published and novel sequences.

We began by downloading human and chicken reference mRNA sequences for all four melatonin genes from GenBank (Underlying data, Supplementary Tables S2–S5 [ Emerling *et al*., 2021]). The protein-coding sequence of each mRNA was BLASTed (megablast) against human and chicken genome assemblies in NCBI’s (National Center for Biotechnology Information) WGS (Whole Genome Shotgun) database. For each gene, we downloaded a contiguous sequence that included all of the mRNA coding exons and flanking sequence, imported the sequences into Geneious v. 2019.2.3 ( Kearse *et al*., 2012), and aligned each mRNA to its corresponding WGS contig hits using MUSCLE v. 3.5 ( Edgar, 2004). We then used the human and chicken assembly-derived sequences as the references for obtaining orthologs in mammals and crocodylians, respectively.

For whole genome assemblies, we BLASTed our reference sequences against assemblies in external and imported databases (Underlying data, Supplementary Table S1 [ Emerling *et al*., 2021]) using intermediate sensitivity settings (e.g., discontiguous megablast on NCBI). When obtaining *AANAT* sequences, the whole reference sequence was used as a probe in BLAST searches and short read mapping. *ASMT* sequences were obtained using a mixture of single exons plus flanking DNA and whole gene reference sequences due to the relatively large size of the gene and small exons. In the case of some *ASMT* sequences, we queried NCBI’s annotated scaffolds directly (RefSeq). *MTNR1A* and *MTNR1B* both have two coding exons separated by a large intron, so to avoid incompatible homology issues when attempting to align the introns, we typically designed separate probes with flanking sequences for the two coding exons. If we ever failed to recover sequences for a species, we used mRNA sequences and annotated gene predictions, plus newly-assembled sequences, as reference sequences for BLAST and mapping, especially from close relatives. Sequences derived from genome assemblies that contain long stretches of Ns can cause issues with alignments, so we trimmed any such instances to ten Ns.

Short reads were obtained from both published (NCBI, Sequence Read Archive) and novel sources. To generate novel sequences for multiple crocodylians ( *Caiman latirostris*, *Crocodylus niloticus*, *Mecistops* sp., *Melanosuchus niger*, *Osteolaemus tetraspis*, *Paleosuchus palpebrosus*, *P. trigonatus*, *Tomistoma schlegelii*), high quality DNA was extracted from tissue using the Qiagen DNeasy kit and quantified through Qubit. In preparation for library construction, 200ng of DNA were sheared with a Covaris S220 for an average size of 500 bp and used as input for the Illumina Neoprep automated library construction instrument. Constructed libraries were pooled and sent to the New York Genome Center for sequencing on HiSeq X system paired-end 150 bp reads.

We imported short reads into Geneious and mapped them to reference sequences (exon + flanking) that were taxonomically close (i.e., same genus, family or order) using intermediate sensitivity settings (medium-low sensitivity). For each set of probes, we performed an initial mapping run to expedite sequence capture, followed by a remapping of the captured reads with the “Fine Tuning” option set to iterate up to five times. Mapped short reads were examined by eye and trimmed of nonhomologous sequences likely derived from adapters or sequencing errors.

For dN/dS analyses in crocodylians, we obtained sauropsid outgroup comparison sequences derived from predicted gene models only (Underlying data, Supplementary Tables S2–S5 [ Emerling *et al*., 2021]), not constructing any such outgroup sequences from assemblies. We BLASTed (discontiguous megablast) each *Alligator mississippiensis* ortholog against NCBI’s nucleotide collection, restricting searches to Squamata, Testudinata and Aves, respectively.

Upon obtaining the mammalian and sauropsid gene sequences, each sequence was imported into Geneious and aligned to its respective probe (i.e., human or chicken whole gene sequence) using MUSCLE. When working with different clades, alignments for each species in that clade were performed successively to provide anchoring and improve subsequent alignments, followed by manual adjustment (see clades in the underlying data, Supplementary Tables S1–S5 [ Emerling *et al*., 2021]). After creating each clade-specific alignment (Underlying data, Supplementary Dataset S1 [ Emerling *et al*., 2021]), we examined the sequences for frameshift indels, splice donor mutations, and splice acceptor mutations. After excising the introns, the coding sequence was translated to search for start codon mutations, premature stop codons, and ancestral stop mutations (Underlying data, Supplementary Tables S2–S5 [ Emerling *et al*., 2021]).

Finally, following the completion of our analyses, a whole genome assembly (WGS) was released for the sirenian Steller’s sea cow ( *Hydrodamalis gigas*). The high similarity to other sirenian gene sequences (pairwise comparison with *Dugong dugon*: *AANAT*: 98%; *ASMT*: 97.4%; *MTNR1A*: 96%; *MTNR1B*: 96.6%) allowed us to positively identify these genes and characterize their functionality, but we did not include them in the evolutionary analyses.

### Evolutionary analyses

Following examination of each clade-specific alignment, global alignments for each gene were assembled to perform phylogenetic (RAxML) and selection pressure (PAML) analyses, respectively (Underlying data, Supplementary Datasets S2–S8 [ Emerling *et al*., 2021]). All stop and incomplete codons were replaced with gaps, as were any codons in individual sequences that were difficult to align with confidence. RAxML analyses were performed with v. 8.2.12 ( Stamatakis, 2014) on CIPRES (RAxML-HPC2 on XSEDE) ( Miller *et al*., 2010), using the default parameters (GTR-CAT), and executing 500 bootstrap replications.

We performed dN/dS ratio analyses using codeml in PAML ver. 4.8 ( Yang, 2007) to test whether genes that appear to be pseudogenized show evidence of undergoing relaxed selection. The mammalian tree topology for these analyses (Underlying data, Supplementary Dataset S9 [ Emerling *et al*., 2021]) was largely derived from a single phylogeny ( Emerling *et al*., 2015), with cetacean, carnivoran, xenarthran and talpid relationships obtained from additional sources ( Allio *et al*., 2021; Gibb *et al*., 2016; He *et al*., 2017; McGowen *et al*., 2020). In some cases, relationships for certain mammals were not resolved in the reference trees, but confamilials and/or congeners were present, allowing us to confidently place such taxa in the phylogeny. For example, our primary reference tree does not include *Desmodus rotundus*, but other phyllostomid species are present. Therefore, *D. rotundus* was positioned in the phyllostomid portion of the PAML tree topology. After setting the topology, we executed one-ratio branch models with one of three codon frequency models (1/61 each, F1X4, F3X4) and used the Akaike information criterion to select the best-fitting codon frequency model for each gene (Underlying data, Supplementary Table S6 [ Emerling *et al*., 2021]).

To test whether inferred pseudogenes are under relaxed selection, we employed branch tests on a series of nested models relative to a ‘master’ model to estimate whether ω is elevated on relevant branches. To construct the ‘master’ models, we used an approach similar to that employed by Meredith *et al*. (2009), categorizing branches as either functional, pseudogenic, mixed/transitional or pre-mutation. ‘Functional’ branches are those for which there is no evidence for gene inactivation, and there is no expectation that the gene is likely to become a pseudogene. All ‘functional’ branches were grouped together for a single ω, were expected to be consistent with purifying selection (ω < 1) and therefore were treated as the background with which to compare the remaining three branch categories. ‘Pseudogenic’ branches are those which post-date a branch on which a gene is inferred to have become a pseudogene. For example, if we mapped an inactivating mutation to the stem cetacean branch, all of the descendant branches within crown Cetacea were grouped together, and a single ω was estimated for this set of branches. In such cases, the dN/dS is expected to approach or be under relaxed selection (ω = 1, higher than functional category). ‘Mixed/transitional’ branches are those on which a gene is inferred to have become a pseudogene, due to one or more inactivating mutations being mapped to that branch. It is therefore suspected to have a mixed history, transitioning from a functional gene to a pseudogene. Depending on how early or late this transition has occurred, the ω is expected to be highly similar to the functional category (more recent inactivation), very similar to the pseudogene category (very early inactivation) or in between these two extremes. Finally, the ‘pre-mutation’ branch category represents instances in which we found no positive evidence of pseudogenization on a branch, but external evidence suggests that relaxed selection may have occurred. Such pre-mutation branches could be designated due to multiple immediate descendant lineages having inactivating mutations and/or one or more other key genes being inactivated. As an example of the former, if a gene is inferred to have been pseudogenized within sloths, armadillos and anteaters separately, given that these all belong to the xenarthran clade, we would designate the branches prior to the mixed/transitional branches of these subclades as ‘pre-mutation’ branches. As an example of the latter, if a taxon had one or both melatonin synthesis genes (e.g., *Fukomys damarensis*) and/or both melatonin receptor genes (e.g., *Heterocephalus glaber*) inactivated, but no evidence of pseudogenization in the remaining genes, we would categorize these branches as ‘pre-mutation’.

As we were not interested in average selection patterns across all branches within a particular category, except the background (functional) branches, we allowed each pre-mutation branch, each mixed/transitional branch and each set of pseudogene branches that post-dated an inactivation event to have their own ω estimates. This allowed us to test whether unique historical instances of putative gene inactivation were consistent with relaxed selection, rather than obscuring the signal by ‘averaging’ pre-mutation, mixed/transitional and pseudogene branches, respectively, across the tree.

After setting these branch categories, this ‘master’ model (Extended data, Supplementary Figures S1–S4 [ Emerling *et al*., 2021]), was calculated for each gene and used as the basis for which to compare subsequent nested models (Underlying data, Supplementary Tables S7–S10 [ Emerling *et al*., 2021])). Specifically, if higher than the background in the master model, we tested whether each set of pseudogene category branches, each mixed/transitional branch and each pre-mutation branch was distinguishable from the background (functional) ω and/or from the neutral ω value of 1 (expectation for pseudogene branches). In such cases, we used the master model for each gene that included all branch categories (Extended data, Supplementary Figures S1–S4 [ Emerling *et al*., 2021]), and would change only one branch of interest to be fixed as being part of the background or 1. We then compared each of the nested models to the master model using likelihood ratio tests to determine which models better fit the data for a branch of interest. In these instances, the models in which branch(es) were fixed as the background or 1 are considered the null model, with the master model being the alternative model, i.e., we tested whether a free estimate of ω for a foreground branch is statistically distinguishable from the background and/or 1. Finally, given the number of comparisons we accumulated, we ran Holm-Bonferroni corrections for multiple testing ( Gaetano, 2018; Holm, 1979) (Underlying data, Supplementary Tables S7–10 [ Emerling *et al*., 2021]).

We performed separate dN/dS ratio analyses for crocodylian melatonin genes by comparing their pattern of molecular evolution to those of 19 other sauropsids (five squamates, five turtles, nine birds). The topology for our analyses (Underlying data, Supplementary Dataset S9 [ Emerling *et al*., 2021]) is a composite derived from separate phylogenies for crocodylians ( Hekkala *et al.,* 2021), squamates ( Zheng & Wiens, 2016), turtles ( Pereira *et al.*, 2017) and birds ( Prum *et al.*, 2015). As above, we first chose the codon frequency model for each gene by running one ratio models and determining the best fit using the Akaike information criterion (Underlying data, Supplementary Table S6 [ Emerling *et al*., 2021]). Then we ran a three-ratio model, estimating ω separately for crown Crocodylia, stem Crocodylia and the background, and compared its fit to a one ratio model using a likelihood ratio test (Underlying data, Supplementary Table S11 [ Emerling *et al*., 2021]).

### Melatonin gene expression

In order to test for evidence of active transcription of the melatonin synthesis genes in a crocodylian, we analyzed published RNA sequencing data from 22 sequencing experiments on tissues in a juvenile *Alligator mississippiensis* ( St John *et al*., 2012). We BLASTed (megablast) the protein-coding regions of the *A. mississippiensis AANAT* and *ASMT* genes against short reads derived from mRNA sequencing in NCBI’s Sequence Read Archive (Underlying data, Supplementary Table S12 [ Emerling *et al*., 2021]), imported the sequences into Geneious, and mapped them to the *A. mississippiensis* references using the low sensitivity setting.

## Results

**Figure 1.**
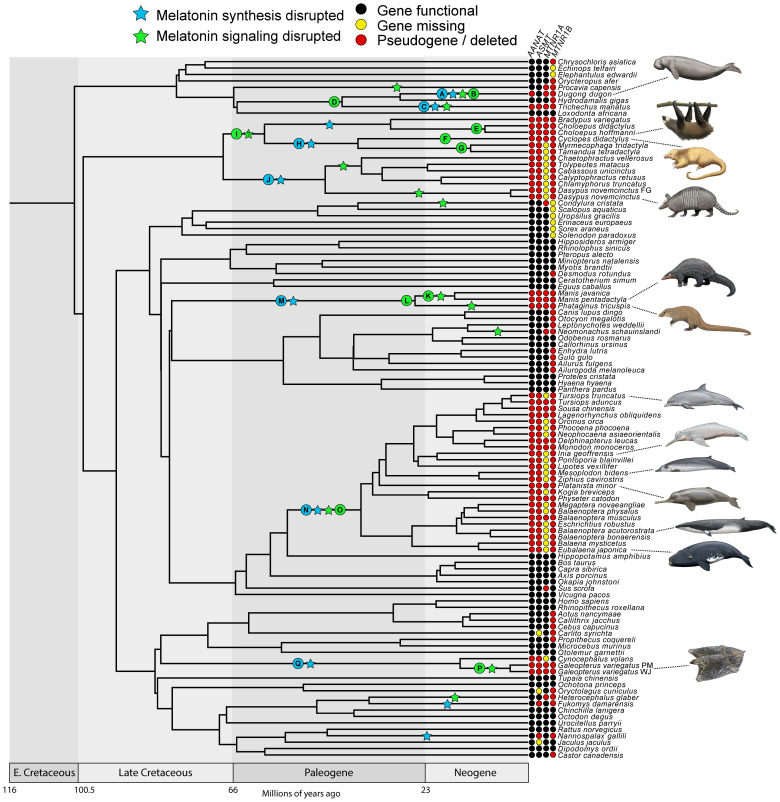
Timetree showing patterns of melatonin gene retention and loss across the placental mammals examined in this study. “Melatonin synthesis disrupted” indicates *AANAT* and/or *ASMT* is inferred to have been inactivated on the associated branch. “Melatonin signaling disrupted” indicates *MTNR1A* and *MTNR1B* are both inferred to have been inactivated on the associated branch, or one gene was lost on an earlier branch and the second was inactivated on the associated branch. Note that the stars are arbitrarily placed in the middle of branches and do not correspond to a precise timing for gene loss. Letters on stars and nodes correspond to letters in Figure 2. References for topology in Materials and Methods. Divergence dates in the figure derived from multiple references ( Gibb *et al*., 2016; Kumar *et al*., 2017; McGowen *et al*., 2020; Meredith *et al*., 2011; Springer *et al*., 2015). Paintings by Carl Buell, copyright John Gatesy.

**Figure 2.**
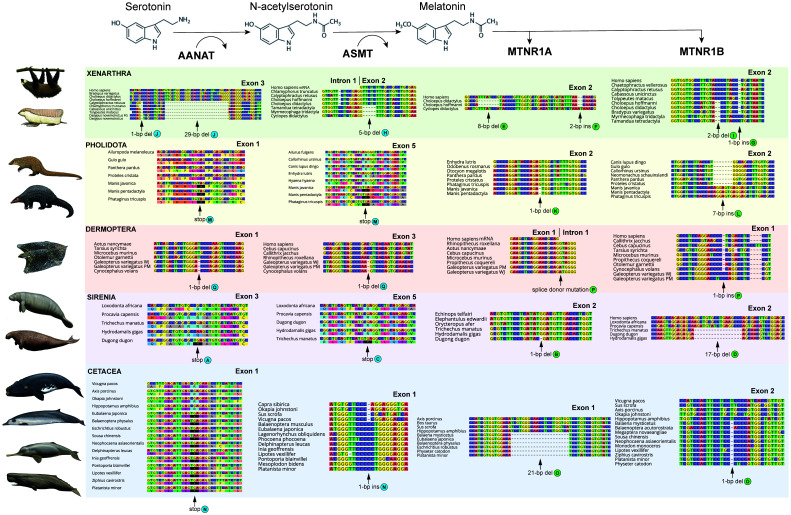
Sampling of inactivating mutations in melatonin pathway genes from five clades of placental mammals. Each column of DNA and protein sequence alignments corresponds to a bolded protein in the melatonin pathways towards the top of the figure. Letters after mutations correspond to letters in the timetree in Figure 1. Ins = insertion; del = deletion; stop = premature stop codon. Paintings by Carl Buell, copyright John Gatesy.

**Figure 3.**
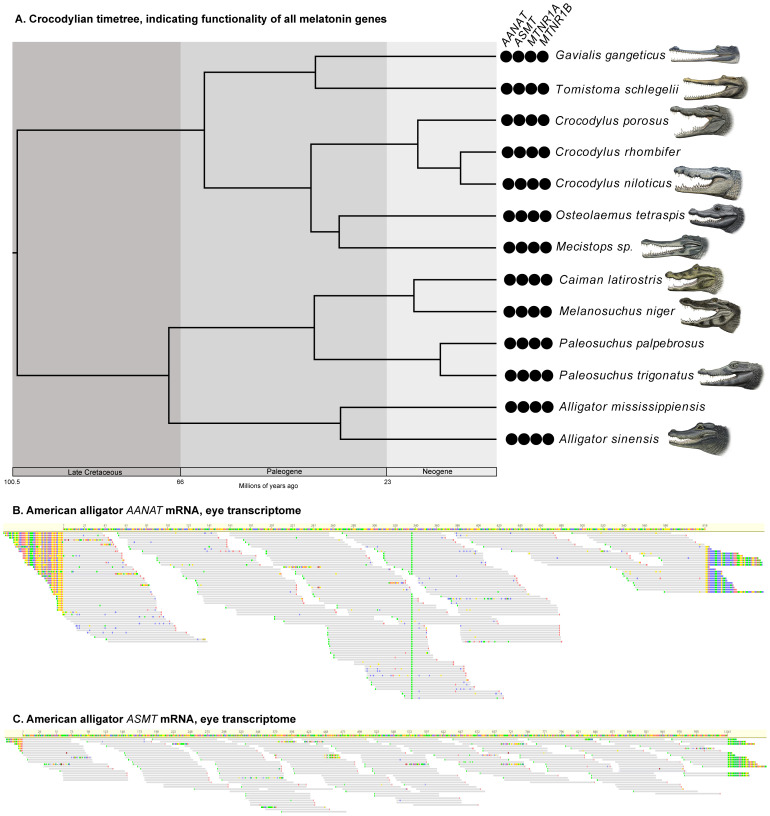
Melatonin genes in crocodylians. **A**. Timetree depicting relationships and divergence times of crocodylians ( Hekkala *et al.*, 2021) examined in this study. The black circles indicate retention of functional orthologs of the genes. Paintings by Carl Buell, copyright John Gatesy. **B**. mRNA short read from an American alligator eye mapped to a reference *AANAT*. **C**. mRNA short reads from an American alligator eye mapped to a reference *ASMT*.

We found evidence that all xenarthrans, all pangolins, and all cetaceans have had both their melatonin synthesis ( *AANAT*, *ASMT*) and melatonin signaling ( *MTNR1A*, *MTNR1B*) genes disrupted via the accumulation of inactivating mutations and whole gene deletions ( Figure 1 & Figure 2; Underlying data, Supplementary Tables S2–S5 [ Emerling *et al*., 2021]). Among sirenians, the West Indian manatee ( *Trichechus manatus*) and dugong ( *Dugong dugon*) have inactivated *AANAT*, *MTNR1A* and *MTNR1B*, with the former being heterozygous for an *ASMT* pseudogene (Underlying data, Supplementary Table S13 [ Emerling *et al*., 2021]) and the latter retaining an intact *ASMT*, whereas the Steller’s sea cow ( *Hydrodamalis gigas*) only has an inactivated *MTNR1B*. By contrast, all 13 crocodylians investigated retain intact melatonin genes ( Figure 3; Underlying data, Supplementary Tables S2–S5 [ Emerling *et al*., 2021]), which show evidence of being under purifying selection (Underlying data, Supplementary Table S11 [ Emerling *et al*., 2021]). Furthermore, we found that *AANAT* and *ASMT* are both expressed in the American alligator, particularly within the eye (Underlying data, Supplementary Table S12 [ Emerling *et al*., 2021]). We also found evidence for the complete disruption of both melatonin gene pathways (i.e., synthesis, signaling) in two dermopteran (colugo) species ( *Galeopterus variegatus* Peninsular Malaysia, *G. variegatus* West Java; considered distinct cryptic species by Mason *et al.*, 2016), with a third ( *Cynocephalus volans*) possibly only retaining a functional *MTNR1B* ( Figure 1 & Figure 2; Underlying data, Supplementary Tables S2–S5 [ Emerling *et al*., 2021]). The melatonin synthesis gene ( *ASMT*) is also inactivated in three subterranean rodents ( *Nannospalax galili*, *Spalax carmeli*, *Fukomys damarensis*), and both melatonin receptor genes appear to be inactivated in hyraxes ( *Procavia capensis*, *Heterohyrax brucei*), a subterranean mole ( *Condylura cristata*) and mole-rat ( *Heterocephalus glaber*), and a seal ( *Neomonachus schauinslandi*) (Underlying data, Supplementary Tables S3–S5, S13 [ Emerling *et al*., 2021]).

The pseudogene status of nearly all of these genes is supported by the positive identity of the genes as orthologs inferred by RAxML analyses (Extended data, Supplementary Figures S5–S7 [ Emerling *et al*., 2021]), the absence of functional paralogs (Extended data, Supplementary Figures S5–S7; Underlying data, Supplementary Tables S2–S5 [ Emerling *et al*., 2021]), presence of shared inactivating mutations ( Figure 2; Underlying data, Supplementary Tables S2–S5, S13; Extended data, Supplementary Figures S8–S12 [ Emerling *et al*., 2021]), the mapping of short reads (Underlying data, Supplementary Table S13; Extended data, Supplementary Figures S9–S14 [ Emerling *et al.,* 2021]) and dN/dS ratio estimates suggestive of extensive relaxed selection (Extended data, Supplementary Figures S15–S18; Underlying data, Supplementary Tables S7–S10 [ Emerling *et al*., 2021]). Specifics are discussed below.

### Crocodylia

We were able to obtain whole or partial genes for all 13 crocodylians we examined, and none of the recovered sequences presented evidence of gene inactivation. Furthermore, dN/dS ratio analyses strongly suggest that all four melatonin genes are under purifying selection within crown Crocodylia ( *AANAT*: background ω = 0.1, crown Crocodylia ω = 0.1; *ASMT*: background ω = 0.2, crown Crocodylia ω = 0.15; *MTRN1A*: background ω = 0.09, crown Crocodylia ω = 0.12; *MTNR1B*: background ω = 0.11, crown Crocodylia ω = 0.13; Underlying data, Supplementary Table S11 [ Emerling *et al*., 2021]). Out of 22 published RNA sequencing experiments on a juvenile *Alligator mississippiensis* (Underlying data, Supplementary Table S12 [ Emerling *et al*., 2021]), five yielded reads that mapped to *AANAT* and/or *ASMT*: eye (both), ovary ( *ASMT*), pooled (intracoelomic fat body, scute muscle, trachea, cloacal gland, gastralia; both), stomach ( *AANAT*) and thalamus ( *ASMT*). The ovary, stomach and thalamus yielded only two reads each, the pooled experiment yielded 27 for *AANAT* and 22 for *ASMT*, and the eye had 251 for *AANAT* and 207 for *ASMT*. The mapped reads for the eye experiment encompassed the entire genes ( Figure 3), strongly suggesting that the melatonin synthesis genes are expressed in one or more ocular tissues.

### Xenarthra

For *AANAT*, sloths (Folivora) share a mutated start codon (ACA), splice donor mutation (GT to GG) in intron 1, 1-bp deletion in exon 2, and two frameshift indels in exon 3. All anteaters (Vermilingua) possess a start codon mutation (GTG or TTG), with six additional inactivating mutations shared between *Myrmecophaga* and *Tamandua*. Armadillos (Cingulata) share a 1-bp deletion in exon 1, and four frameshift indels in exon 3 ( Figure 2). In *ASMT*, there is a putative 1-bp insertion in exon 6 shared by two-toed sloths ( *Choloepus* spp.), two chlamyphorid armadillos ( *Calyptophractus*, *Chlamyphorus*), and a dasypodid armadillo ( *Dasypus novemcinctus* French Guiana). This region is missing in all other xenarthran sequences (i.e., deleted or not assembled), and the alignment is admittedly ambiguous. Furthermore, in all xenarthrans we examined, exon 5 appears to be absent and the ancestral stop codon (i.e., the stop codon common to all other species examined) is mutated, although it differs among sloths (TAT), anteaters (TGT or TGG), and armadillos (CGT or TGT). Beyond these, shared inactivating mutations are present among the anteaters (5-bp deletion, exon 2 [ Figure 2]; two frameshift indels, exon 8), sloths (1-bp deletion, exon 3; 4-bp insertion, exon 7), and armadillos (1-bp deletion, exon 6), respectively. We were unable to obtain *MTNR1A* in most xenarthrans, possibly due to a whole gene deletion, although the assemblies were not complete enough to verify this via a synteny analysis. Nonetheless, the silky anteater ( *Cyclopes didactylus*) and sloths retain one or more *MTNR1A* pseudogenes ( Figure 2). Sloths appear to have multiple paralogs, the identities of which are difficult to tease out, with separate exons being found on separate contigs, but the pattern suggests all are probably inactivated. For *MTNR1B*, a 2-bp deletion in exon 2 is shared by sloths and anteaters (Pilosa; Figure 2). Among armadillos, a single 1-bp deletion (exon 1) is shared by dasypodids, and five inactivating mutations are shared among chlamyphorid armadillos (two in exon 1; three in exon 2).

From dN/dS ratio analyses, we found evidence of shifts in selection pressures consistent with ancient pseudogenization of these genes. Estimates in which key xenarthran branches had statistically elevated ω values were found for *AANAT* (background: 0.36; stem Folivora: 1.57*; crown Folivora: 0.71; stem Vermilingua: 2.81*; crown Vermilingua: 1.71*; stem Cingulata: 7.33*; crown Cingulata: 1.34*), *ASMT* (background: 0.25; crown Folivora: 0.56; crown Vermilingua: 1.07*; crown Cingulata: 0.91*; stem Cingulata: 1.06*), *MTNR1A* (background: 0.17; crown *Choloepus*: 1.06; stem *Choloepus*: 0.76*; *Cyclopes didactylus*: 1.11*) and *MTNR1B* (background: 0.32; crown Pilosa: 1.18*; crown Chlamyphoridae: 0.79*; stem Dasypodidae: 2.36*; * indicates model comparison remains statistically significant after Holm-Bonferroni correction, here and below). We also ran a model for *ASMT* in which crown Xenarthra was given a single ω, under the assumption that it was inactivated in the stem lineage (see above). This ω was estimated as 0.79, and when compared to a model in which the crown Xenarthra ω was fixed at 1, the former was a better fit for the data than the latter. As such, this result seems to be inconsistent with a stem xenarthran inactivation of *ASMT*.

### Pholidota

For pangolins, all three species share two premature stop codons in *AANAT* (exons 1 and 2), another in exon 5 of *ASMT*, and four inactivating mutations in *MTNR1B* ( Figure 2). Exon 1 of *MTNR1A* is missing for both *Manis* spp., preventing comparisons with *Phataginus tricuspis*, but the former two share a 1-bp deletion in exon 2 ( Figure 2). Moreover, dN/dS analyses provide further evidence of pseudogenization with elevated ω values for *AANAT* (background: 0.36; crown Pholidota: 0.62), *ASMT* (background: 0.25; crown Pholidota: 0.72*), *MTNR1A* (background: 0.17; crown *Manis*: 1.11*; stem *Manis*: 3.35*; *Phataginus*: 1.43*; stem Pholidota: 0.35), and *MTNR1B* (background: 0.32; crown Pholidota: 1.51*; stem Pholidota: 1.14*).

### Cetacea

Inactivation of the melatonin genes in cetaceans has already been reported in two recent studies ( Huelsmann *et al*., 2019; Lopes-Marques *et al*., 2019), although we have expanded on these results by increasing the taxon sampling from 12 to 25 species and eight to 13 families ( Figure 1), as well as analyzing the selection patterns of this clade. As in previous studies, we found shared inactivating mutations, including a premature stop codon in exon 1 of *AANAT* (all 25 species), a 1-bp insertion in exon 1 of *ASMT* (21 species, mysticetes + odontocetes), and a 1-bp deletion in exon 2 of *MTNR1B* (17 species, mysticetes + odontocetes) ( Figure 2). For *MTNR1A*, almost all toothed whales (Odontoceti) completely lack the gene in their assemblies, with the exceptions of pseudogenes in *Platanista minor* and *Physeter catodon*, whereas all examined baleen whales (Mysticeti), *Platanista* and *Physeter* lack exon 2. Using contiguous assemblies from one mysticete and five odontocetes, we confirmed via a synteny analysis that exon 2 is absent (Extended data, Supplementary Figure S8 [ Emerling *et al.*, 2021]), suggesting a whole exon deletion occurred on the stem Cetacea branch. Furthermore, an unusual 21-bp deletion in exon 1 shared by odontocetes and mysticetes may represent an in-frame disabling mutation ( Figure 2). dN/dS analyses are consistent with relaxed selection in this clade, with statistically elevated ω values in *AANAT* (background: 0.36; crown Cetacea: 0.99*; stem Cetacea: 3.2), *ASMT* (background: 0.25; crown Cetacea: 0.93*; stem Cetacea: 0.7), *MTNR1A* (background: 0.17; stem Cetacea: 1.33) and *MTNR1B* (background: 0.32; crown Cetacea: 1.02*; stem Cetacea: 0.64).

### Sirenia

For sirenians, shared inactivating mutations are less common, existing only for *MTNR1B* (exon 2: 14-bp deletion, 17-bp deletion [ Figure 2], ancestral stop mutation TAC or TGC). Outside of this gene, inactivating mutations are only found in the manatee ( *AANAT*: two; *ASMT*: one; *MTNR1A*: two) and dugong ( *AANAT*: two; *MTNR1A*: two) ( Figure 2), although the sole inactivating mutation in the manatee *ASMT* appears to be polymorphic based on short read data (Extended data, Supplementary Figure S12 [ Emerling *et al*., 2021]). Some of the manatee mutations were previously reported ( Huelsmann *et al*., 2019; Lopes-Marques *et al*., 2019). Here, dN/dS analyses only provide evidence of relaxed selection in *MTNR1A* (background: 0.17; stem Sirenia: 0.48; *Dugong*: 0.53*; *Trichechus*: 1.95*) and *MTNR1B* (background: 0.32; crown Sirenia: 1.95*).

### Dermoptera

Both melatonin synthesis genes have shared inactivating mutations across all three colugos: exon 1 of *AANAT* and exon 3 of *ASMT* each have shared 1-bp deletions, ( Figure 2). We were unable to obtain *MTNR1A* for *Cynocephalus volans*, but both *Galeopterus* species have a splice donor mutation (AG to AT) in the intron ( Figure 2). Similarly, we were unable to assemble exon 2 of *MTNR1B* for *C. volans*, and exon 1 appears intact, but *Galeopterus* spp. share a 1-bp insertion (exon 1; Figure 2) and a premature stop codon (exon 2). Furthermore, dN/dS estimates provide evidence of relaxed selection in *AANAT* (background: 0.36; crown Dermoptera: 0.77), *ASMT* (background: 0.25; crown Dermoptera: 0.6) and *MTNR1A* (background: 0.17; crown *Galeopterus*: 0.81*; stem *Galeopterus*: 0.64*).

### Other placental mammals

Beyond these taxa, *AANAT* is present as two to three paralogs in multiple non-cetacean cetartiodactyls, with one paralog sometimes being a pseudogene, a finding corroborated by a recent study ( Yin *et al*., 2021). However, at least one gene is always intact in all non-cetacean cetartiodactyls that we examined. *ASMT* is a pseudogene in three subterranean rodents ( *Fukomys damarensis* [Bathyergidae]; *Nannospalax galili*, *Spalax carmeli* [Spalacidae]), *MTNR1A* is inactivated in two hyraxes ( *Procavia capensis*, *Heterohyrax brucei*, Hyracoidea), a monk seal ( *Neomonachus schauinslandi*), pig ( *Sus scrofa*), talpid mole ( *Condylura cristata*), and the naked-mole rat ( *Heterocephalus glaber*), and *MTNR1B* is a pseudogene or likely deleted (i.e., negative BLAST results) in a host of other species, including seven afrotherians, eight carnivorans, one bat, all six examined eulipotyphlans, one lagomorph, five primates, and five rodents we examined ( Figure 1). The few examples of shared inactivating mutations we found in these other species include a 4-bp insertion in exon 6 of *ASMT* in two spalacids (Extended data, Supplementary Figure S9 [ Emerling *et al*., 2021]), a 1-bp deletion in exon 2 of *MTNR1A* (Extended data, Supplementary Figure S10 [ Emerling *et al*., 2021]) and three inactivating mutations in *MTNR1B* shared by two hyraxes, and a 1-bp insertion in exon 2 of *MTNR1B* for two platyrrhine monkeys ( *Callithrix jacchus*, *Cebus capucinus*). Here also, dN/dS ratio analyses suggest that many of these pseudogenes are indeed under relaxed selection based on statistically elevated ω values.

## Discussion

### Melatonin genes inactivated in many mammals, but functional in crocodylians

Here we reported evidence that xenarthrans, pangolins, cetaceans and some sirenians have lost the capability to synthesize and bind melatonin via the traditional pathway found in vertebrates, coinciding with the ostensible absence of a pineal gland in these taxa. This builds upon recent studies on cetaceans and the West Indian manatee ( Huelsmann *et al*., 2019; Lopes-Marques *et al*., 2019), demonstrating the surprising extent of the degradation of these genes. We hypothesized that despite the pineal gland’s apparent absence in xenarthrans, pangolins, sirenians, cetaceans and crocodylians, the genes underlying the production and signaling of melatonin would remain intact, given the widespread effects of melatonin in vertebrates and evidence of circulating melatonin in these taxa ( Firth *et al*., 2010; Funasaka *et al*., 2011; Harlow *et al*., 1981; Panin *et al*., 2012; Roth *et al*., 1980). Indeed, this appears to be the case for crocodylians, in which all 13 species we investigated, representing all major lineages, possess intact orthologs of *AANAT*, *ASMT*, *MTNR1A* and *MTNR1B*. Given the presence of serum melatonin in at least some crocodylians ( Firth *et al*., 2010; Roth *et al*., 1980), this suggests that either the pineal gland is intact but difficult to isolate and/or extra-pineal sources of melatonin exist. In support of the latter hypothesis, we found evidence of *AANAT* and *ASMT* expression in the eyes of the American alligator ( *Alligator mississippiensis*). Furthermore, a previous study found that the gene encoding a pineal opsin pigment is a pseudogene in crocodylians ( Emerling, 2017b), potentially revealing a shift in the source of melatonin from the pineal gland to the eye, following the regression of the former.

By contrast, most major melatonin pathway genes are pseudogenized in pineal-less mammals. Researchers have varied in their reports on the presence versus absence of pineal glands in cetaceans ( Behrmann, 1990; Holzmann, 1991; Lyamin *et al*., 2008; McFarland *et al*., 1969; Oelschläger *et al*., 2008; Panin *et al*., 2012) and work on sirenians appears to be inconsistent, with some studies suggestive of a minute pineal gland and others of its complete absence ( Chapman, 1875; Murie, 1872; Ralph *et al*., 1985). A recent anatomical study failed to find a pineal gland in the white-bellied pangolin ( *Phataginus tricuspis*), although the absence was attributed to an error in preparing the brain for study ( Imam *et al*., 2019). Despite these inconsistencies, a regressed, if not completely absent, pineal gland largely predicts the disappearance of the canonical melatonin pathways, at least in mammals. Furthermore, we found evidence of melatonin synthesis inactivation in colugos, suggesting that these species may also possess a regressed pineal gland, or may lack it entirely. Although one study found a diencephalon in colugos comparable in relative size to gliding rodents and bats ( Pirlot & Kamiya, 1982), a review of pinealocytes in mammals noted the lack of research specifically on the pineal in Dermoptera ( Bhatnagar, 1992).

While extra-pineal melatonin may reconcile the patterns of circulating melatonin and the putative absence of a pineal gland in crocodylians, it cannot explain this same phenomenon in the nine-banded armadillo ( Harlow *et al*., 1982; Harlow *et al*., 1981) and bottlenose dolphins ( Funasaka *et al*., 2011; Panin *et al*., 2012). Possible sources for the serum melatonin detected in these taxa may derive from an unknown alternative pathway ( Tan *et al*., 2016), dietary sources ( Hattori *et al*., 1995; Peuhkuri *et al*., 2012; Reiter *et al*., 2005), or the organism’s microbiome ( Hardeland & Poeggeler, 2003). However, the functional significance of serum melatonin may be obviated by the absence of melatonin receptors in these species, presumably preventing any contribution to circadian signaling in the body’s tissues. Despite this, given that recent discoveries have shown that presenting as a pseudogene does not always indicate that all biological function is lost ( Cheetham *et al.,* 2020), there remains a possibility that these apparently dysfunctional genes are able to contribute to melatonin metabolism in some unknown fashion.

### Ancient loss of melatonin genes in placental mammals

Our results strongly suggest that melatonin synthesis and signaling have been abolished within multiple placental mammal lineages for extensive periods of evolutionary time. The most ancient of these may be in Xenarthra, given our evidence of possible *ASMT* and *MTNR1A* inactivation on the stem branch. Crown Xenarthra arose roughly 68 million years ago (mya) ( Gibb *et al*., 2016), potentially meaning that the loss of melatonin synthesis and possibly some signaling took place near the K/Pg boundary, when non-avian dinosaurs went extinct and placental mammals began to radiate. However, given some ambiguity in the evidence for stem inactivation of *ASMT* (see results), and the absence of sequences of *MTNR1A* for most species of xenarthrans, convergent loss remains a strong possibility. Despite this, additional shared mutations in *AANAT* and *ASMT* suggest that the components for melatonin synthesis were disrupted prior to the origin of armadillos (45 mya), sloths (31 mya), and anteaters (38 mya), respectively. In addition, *MTNR1B* was likely pseudogenized prior to the sloth / anteater split (59 mya), and the origin of chlamyphorid armadillos (37 mya). In pangolins, shared mutations in *AANAT*, *ASMT*, and *MTNR1B* suggest complete loss of melatonin synthesis and at least some melatonin signaling prior to the origin of this clade 25 mya ( Meredith *et al*., 2011). For colugos, *AANAT* and *ASMT* were likely inactivated prior to the origin of crown Dermoptera, indicating the absence of melatonin synthesis for at least 15 million years ( Mason *et al*., 2016). There are contrasting patterns among the aquatic taxa, with cetaceans having convincingly lost all four genes prior to their origin 37 mya ( McGowen *et al*., 2020), whereas for sirenians, we only have positive evidence of *MTNR1B* inactivation in the stem lineage at least 42 mya ( Springer *et al*., 2015). Subsequent parallel pseudogenization events occurred in other melatonin-related genes for the manatee and dugong, but not Steller’s sea cow.

### Causes and consequences of losing the melatonin pathway

The potential significance of the loss of both melatonin synthesis and signaling in multiple clades of mammals should not be understated. To reiterate, melatonin is a ubiquitous biogenic compound found in Eubacteria, unicellular eukaryotes, plants, fungi and animals ( Hardeland & Poeggeler, 2003). While it is unclear if melatonin synthesis has a single origin or evolved independently in several lineages ( Tan *et al*., 2014; Zhao *et al*., 2019), its widespread taxonomic occurrence suggests that it has ancient origins and that natural selection has favored the maintenance of synthesis pathways for perhaps billions of years. Furthermore, after a hypothesized co-option of this potent antioxidant to signal environmental darkness ( Zhao *et al*., 2019), in order to help modulate circadian and circannual physiological processes, melatonin synthesis and signaling would seemingly become indispensable for most vertebrates. As such, it is a challenge to clarify the causes and consequences of losing melatonin pathway genes ( Valente *et al.,* 2021).

Convergent evolution often results from similar selection pressures, which may explain why both cetaceans and some sirenians have lost these genes. Perhaps the unique demands of a fully aquatic lifestyle, such as the need to frequently surface for respiration, needed to be uncoupled from a rhythmic signal of darkness. By contrast, the semi-aquatic sea otter ( *Enhydra lutris*) and pinnipeds have retained their melatonin synthesis genes, although *MTNR1B* is pseudogenized in *E. lutris* and two seals (Phocidae), with one of the phocids also showing evidence of *MTNR1A* inactivation. Perhaps this underlies their intermediate aquatic phenotype, although eight of the 13 carnivorans also present an *MTNR1B* pseudogene. Another example of strong convergent evolution can be seen in xenarthran anteaters and pangolins, both of which have radically modified their feeding apparatus to ingest ants and termites (myrmecophagy). However, other myrmecophagous taxa we examined, including the aardvark ( *Orycteropus afer*), aardwolf ( *Proteles cristatus*), and bat-eared fox ( *Otocyon megalotis*) at most only have *MTNR1B* inactivated.

Regardless of their specific phenotypes, all of these taxa experience fluctuations in light and darkness, so it is unclear as to why loss of such a hormone would be beneficial. By contrast, it seems more logical for melatonin synthesis to be lost while adapting to an environment of nearly complete darkness, in which rhythmic secretions entrained on light patterns may no longer be possible. Multiple lineages of subterranean mammals fit this description, and indeed, we found evidence of *ASMT* pseudogenes in the subterranean rodents *Nannospalax galili*, *Spalax carmeli* (Spalacidae) and *Fukomys damarensis* (Bathyergidae), and inactivation of both receptors in the naked mole-rat ( *Heterocephalus glaber*; Bathyergidae). The latter result had been previously reported in a single individual ( Kim *et al*., 2011), but we confirmed that both genes share the same disabling mutations in a second individual *H. glaber* and are likely under relaxed selection. The fossorial star-nosed mole, *Condylura cristata* (Talpidae), also appears to have dispensed of both melatonin receptors, with *MTNR1A* a pseudogene and *MTNR1B* being completely absent from the assembly. In addition, dN/dS estimates suggest selection on *ASMT* is relaxed in *H. glaber*, a species which also has an atrophied pineal gland ( Quay, 1981), and *MTNR1B* is inactivated in *N. galili*, *F. damarensis* and a golden mole ( *Chrysochloris asiatica*; Chrysochloridae). This may be relevant to the ancient loss of melatonin synthesis and signaling in xenarthrans, given that comparative anatomy and an analysis of genes critical for vision in bright light appear to point to an early subterranean history for this clade ( Emerling & Springer, 2015). Perhaps an extended history underground limited the utility of melatonin synthesis and signaling, and upon emerging from this committed existence in the darkness, their descendants inherited this unusual phenotype. However, a pineal gland that can synthesize melatonin has been reported in at least one spalacid ( Balemans *et al*., 1980), pineal glands are reported to be present in talpids and chrysochlorids ( Legait *et al*., 1976; Pevet, 1974; Pevet & Kuyper, 1978) and the melatonin synthesis genes remain intact for *Chrysochloris asiatica* and *Condylura cristata*.

 One potentially unifying concept for the pattern of pineal gland / melatonin synthesis loss may be related to thermoregulation. Ralph (1975) hypothesized that the size of the pineal gland in vertebrates may be correlated to thermoregulation, tentatively linking pineal gland size to latitude, activity pattern and relative homeothermy. He observed that some of the largest pineal glands belong to species that inhabit higher latitudes, while pointing out that some vertebrates with the smallest or absent pineal glands tend to be restricted to the tropics. Though the comparisons were limited, a better-substantiated pattern was noted with the related parietal eye of squamates. The parietal eye is an organ that is developmentally related to and anatomically linked with the pineal gland, which appears to largely provide information for thermoregulation in ectothermic squamates, possibly through melatonin regulation ( Firth & Kennaway, 1980; Hutchison & Kosh, 1974; Phillips & Harlow, 1981; Stebbins & Eakin, 1958). When comparing the presence or absence of the parietal eye, researchers noted a trend of parietal eye loss in squamates that live near the equator ( Gundy *et al*., 1975). Given that the parietal eye provides information about temperature and light, which largely correlates with the amount of sunlight, and the pineal gland secretes melatonin in darkness, the latitudinal hypothesis may have some validity. At lower latitudes, there is less seasonality; therefore, being able to detect changes in circadian and circannual dark and light cues is plausibly of less adaptive benefit in these regions. Notably, xenarthrans, pangolins, sirenians and colugos live almost exclusively in the tropics; furthermore, aquatic and subterranean habitats provide a buffering effect from temperature fluctuation. These characteristics encompass all taxa we record as lacking melatonin synthesis capabilities, making it a potentially attractive hypothesis.

Significantly, the patterns of melatonin pathway degradation have strong overlap with placental mammals that have lost the capacity for non-shivering thermogenesis (NST). Specifically, xenarthrans, pangolins, cetaceans and sirenians all have inactivated *UCP1*, a gene that facilitates NST ( Gaudry *et al*., 2017). Furthermore, hyraxes (Hyracoidea) and pigs (Suidae) have a *UCP1* pseudogene, hyraxes ( *Procavia capensis, Heterohyrax brucei*) have both melatonin receptor genes inactivated, and the wild boar ( *Sus scrofa*) has an *MTNR1A* pseudogene. Notably, melatonin induces the production of brown adipose tissue ( Heldmaier *et al*., 1981; Heldmaier & Hoffmann, 1974), a major location of NST. Together, these data suggest that the loss of melatonin synthesis may be coupled with the loss of this thermoregulatory tool in these clades, further underscoring a potential link between melatonin pathway loss and changes in thermoregulatory requirements.

## Conclusion

In this study we provided evidence that, in contrast to crocodylians, numerous placental mammals reported to lack a pineal gland also lack the genes necessary for the canonical vertebrate melatonin synthesis and signaling pathways. However, this result seems to raise more questions than answers. Given the pleiotropic nature of melatonin synthesis and signaling genes, which selection pressure(s) could have led to the loss of this seemingly crucial signaling molecule? What are the physiological consequences of this loss? Are there possibly compensatory alternative mechanisms for producing and sensing melatonin? For those species that present serum melatonin, how are they doing so? Does this melatonin function merely as an antioxidant, or does it aid in circadian and circannual signaling via different pathways? Further studies on comparative anatomy, physiology and gene expression in pineal-less taxa and others should shed further light on these challenging questions.

## Data availability

### Underlying data

Zenodo: Genomic evidence for the parallel regression of melatonin synthesis and signaling pathways in placental mammals. https://doi.org/10.5281/zenodo.4894211 ( Emerling *et al*., 2021)

This project contains the following underlying data:

- Supplementary_Dataset_S1.txt: Genomic alignments in fasta format used to determine the pseudogene/functional status of all four melatonin genes in different taxonomic groups.
- Supplementary_Dataset_S2.txt: Alignment of *AANAT* in phylip format used in maximum likelihood phylogenetic reconstruction with RAxML.
- Supplementary_Dataset_S3.txt: Alignment of *ASMT* in phylip format used in maximum likelihood phylogenetic reconstruction with RAxML.
- Supplementary_Dataset_S4.txt: Alignment of *MTNR1A* and *MTNR1B* in phylip format used in maximum likelihood phylogenetic reconstruction with RAxML.
- Supplementary_Dataset_S5.txt: Codon alignments of *AANAT* used in selection pressure analyses with PAML.
- Supplementary_Dataset_S6.txt: Codon alignments of *ASMT* used in selection pressure analyses with PAML.
- Supplementary_Dataset_S7.txt: Codon alignments of *MTNR1A* used in selection pressure analyses with PAML.
- Supplementary_Dataset_S8.txt: Codon alignments of *MTNR1B* used in selection pressure analyses with PAML.
- Supplementary_Dataset_S9.txt: Tree topologies in newick format used in selection pressure analyses with PAML.
- Supplementary_Table_S1.xlsx: List of species examined in this study and the sources of the genes.
- Supplementary_Table_S2.xlsx: Accession numbers and functionality of *AANAT* in species examined.
- Supplementary_Table_S3.xlsx: Accession numbers and functionality of *ASMT* in species examined.
- Supplementary_Table_S4.xlsx: Accession numbers and functionality of *MTNR1A* in species examined.
- Supplementary_Table_S5.xlsx: Accession numbers and functionality of *MTNR1B* in species examined.
- Supplementary_Table_S6.xlsx: Codon frequency model selection.
- Supplementary_Table_S7.xlsx: Results of *AANAT* PAML dN/dS analyses for mammals.
- Supplementary_Table_S8.xlsx: Results of *ASMT* PAML dN/dS analyses for mammals.
- Supplementary_Table_S9.xlsx: Results of *MTNR1A* PAML dN/dS analyses for mammals.
- Supplementary_Table_S10.xlsx: Results of *MTNR1B* PAML dN/dS analyses for mammals.
- Supplementary_Table_S11.xlsx: Results of PAML analyses for sauropsids.
- Supplementary_Table_S12.xlsx: Results of BLASTing and mapping short reads from *Alligator mississippiensis* RNA sequencing experiments.
- Supplementary_Table_S13.xlsx: Supporting data for validating putative inactivating mutations.

### Extended data

Zenodo: Genomic evidence for the parallel regression of the melatonin synthesis and signaling pathways in placental mammals. http://doi.org/10.5281/zenodo.4894211 ( Emerling *et al*., 2021)

This project contains the following extended data:

- Supplementary_Figure_S1.pdf: *AANAT* PAML ‘master model’ showing branch categories, corresponding to “Model 1: 24 ratio” in Supplementary Table S7.
- Supplementary_Figure_S2.pdf: *ASMT* PAML ‘master model’ showing branch categories, corresponding to “Model 2: 24 ratio” in Supplementary Table S8.
- Supplementary_Figure_S3.pdf: *MTNR1A* PAML ‘master model’ showing branch categories, corresponding to “Model 1: 27 ratio” in Supplementary Table S9.
- Supplementary_Figure_S4.pdf: *MTNR1B* PAML ‘master model’ showing branch categories, corresponding to “Model 1: 46 ratio” in Supplementary Table S10.
- Supplementary_Figure_S5.pdf: RAxML *AANAT* gene tree. Numbers at nodes correspond to bootstrap support values.
- Supplementary_Figure_S6.pdf: RAxML *ASMT* gene tree. Numbers at nodes correspond to bootstrap support values.
- Supplementary_Figure_S7.pdf: RAxML *MTNR1A*+ *MTNR1B* tree. Numbers at nodes correspond to bootstrap support values.
- Supplementary_Figure_S8.pdf: supporting data showing the inactivation of *MTNR1A* exon 2 in cetaceans.
- Supplementary_Figure_S9.pdf: supporting data showing the inactivation of *ASMT* in spalacids and *Fukomys damarensis*.
- Supplementary_Figure_S10.pdf: supporting data showing the inactivation of *MTNR1A* in hyracoids and *Cyclopes didactylus*.
- Supplementary_Figure_S11.pdf: supporting data showing the inactivation of *MTNR1A* in sirenians.
- Supplementary_Figure_S12.pdf: supporting data showing the inactivation of *AANAT* in sirenians and a polymorphic premature stop codon in exon 5 of *ASMT* in *Trichechus manatus*.
- Supplementary_Figure_S13.pdf: supporting data showing the inactivation of *MTNR1A* in *Condylura cristata*.
- Supplementary_Figure_S14.pdf: supporting data showing the inactivation of *MTNR1A* in *Phataginus tricuspis*.
- Supplementary_Figure_S15.pdf: PAML *AANAT* results, Model 1: 24 ratio.
- Supplementary_Figure_S16.pdf: PAML *ASMT* results, Model 2: 24 ratio.
- Supplementary_Figure_S17.pdf: PAML *MTNR1A* results, Model 1: 27 ratio.
- Supplementary_Figure_S18.pdf: PAML *MTNR1B* results, Model 1: 46 ratio.

Data are available under the terms of the Creative Commons Attribution 4.0 International license (CC-BY 4.0).
