# Supplementary material for: Genomic evidence for the parallel regression of melatonin synthesis and signaling pathways in placental mammals
Source: Open Res Eur. 2021 Dec 13;1:75. Originally published 2021 Jul 1. [Version 2] doi: 10.12688/openreseurope.13795.2 (PMC7613276; doi:10.12688/openreseurope.13795.2)
Supplement: Peer review history

# Peer review: Reviewer response for version 2

**DOI:** 10.21956/openreseurope.15466.r28164

**Published:** 2022-01-21

**Authors:** Ryan K Schott

**Affiliations:** 1 Department of Biology, York University, York, ON, Canada

The authors have done a nice job addressing the concerns of myself and the other Reviewer. I am generally satisfied with the outcome and believe the paper to be improved.

 While I find the PAML analyses easier to understand now, I still find the approach taken unusual. That is, using one alternate model and comparing it to numerous different null models rather than the other way around (or by successively comparing pairs of more complex models). I can better understand the reasoning behind this now, but there is a potential issue in that each null model employed by the authors has not been shown to itself be a significant improvement over a less complex model. Further, a general comparison among models (eg., using AIC values) was not performed and is not possible since the number of parameters in each model is not shown (as a side note: it would be nice to add these to the tables). With the tests done, there is often no way to distinguish between two 'null models' that place a branch in the background or fix it to w=1.

 The authors indicated they were not interested in average patterns, but I think these could have been useful for the reader to get a broader picture of the patterns and to test for specific differences. In some cases, it is not clear why we would expect differences among lineages. For example, would we expect dn/ds to differ for pseudogenes among lineages?

 That said I think the analysis performed by the authors is sufficient to support their conclusions and am happy to add my approval.

Is the study design appropriate and does the work have academic merit?

Yes

Is the work clearly and accurately presented and does it cite the current literature?

Yes

If applicable, is the statistical analysis and its interpretation appropriate?

Partly

Are all the source data underlying the results available to ensure full reproducibility?

Partly

Are the conclusions drawn adequately supported by the results?

Yes

Are sufficient details of methods and analysis provided to allow replication by others?

Partly

Reviewer Expertise:

molecular evolution, comparative genomics, sensory biology

I confirm that I have read this submission and believe that I have an appropriate level of expertise to confirm that it is of an acceptable scientific standard.

# Peer review: Reviewer response for version 2

**DOI:** 10.21956/openreseurope.15466.r28165

**Published:** 2021-12-14

**Authors:** Mareike C. Janiak

**Affiliations:** 1 School of Science, Engineering and Environment, University of Salford, Salford, UK

I appreciate the authors' thorough responses to both reviews. I am satisfied with the changes made to the manuscript in response to my review and have no further comments.

Is the study design appropriate and does the work have academic merit?

Yes

Is the work clearly and accurately presented and does it cite the current literature?

Yes

If applicable, is the statistical analysis and its interpretation appropriate?

Yes

Are all the source data underlying the results available to ensure full reproducibility?

Yes

Are the conclusions drawn adequately supported by the results?

Yes

Are sufficient details of methods and analysis provided to allow replication by others?

Partly

Reviewer Expertise:

comparative genetics/genomics, dietary adaptations

I confirm that I have read this submission and believe that I have an appropriate level of expertise to confirm that it is of an acceptable scientific standard.

# Peer review: Reviewer response for version 1

**DOI:** 10.21956/openreseurope.14874.r27206

**Published:** 2021-07-20

**Authors:** Ryan K Schott

**Affiliations:** 1 Department of Biology, York University, York, ON, Canada

In this study, the authors examined two melatonin synthesis genes ( *AANAT* and *ASMT*) and two melatonin receptors ( *MTNR1A* and *MTNR1B*) in 110 placental mammals and 13 crocodylians. They hypothesized that species which lack pineal glands, the primary source of melatonin production, would lack functional melatonin synthesis and receptor genes. The authors found support for this hypothesis in placental mammals, but not in crocodylians. In the 13 crocodylians examined, all of which are thought to lack pineal glands, the authors found intact melatonin genes and expression of two of those genes was confirmed with tissue-specific transcriptome sequencing in American alligator, specifically in the eye. In contrast, placental mammals thought to lack pineal glands (xenarthrans, pangolins, sirenians, and whales), as well as colugos and some fossorial mammal lineages, showed evidence for independent inactivation and loss of all four melatonin genes. Many other lineages also showed evidence of inactivation or loss in a smaller number of the genes. The authors speculate that loss of the melatonin synthesis and receptor genes may be associated with changes in thermoregulation and the loss of non-shivering thermogenesis. Overall, the manuscript is very well written, presents new and interesting results, and outlines several important areas for future research. I outline below several areas where the authors could further improve the paper:

- Inference of gene loss and pseudogenization depends on high quality sequencing data and genomic assemblies and apparent loss and pseudogenization could be due to incomplete sequencing/assembly or sequencing/assembly errors. While patterns of shared indels or premature stop codons are particularly convincing, I believe this caveat should still be addressed, especially considering that ~70% of the mammals examined were listed as having at least one pseudogenized or lost gene.
- Further to the point above, the sources of the genomic data used in the study is unclear. The main supplementary table of the genome sources does not provide much meaningful information (e.g., source listed as WGS). Looking through some of the subsequent SI tables accession numbers are given for some of the genes, but others are simply listed as New. Ideally all of the genomes would be made available, but minimally more detail needs to be provided on the genomic data (quality, completeness, etc…) used to obtain the sequences analyzed in the current study. It is difficult to evaluate the robustness of the results or reproduce the study without this.
- It was also unclear whether the transcriptome data were newly sequenced or the analysis was of previously published data. More detail should be provided.
- The explanation of the methodology and approach used in the selection analyses was difficult to follow and perhaps overly complex. The authors mention a nested approach but do not provide much detail. Looking through the supplementary tables of PAML results I do not see nested sets of models or an attempt to find the best fitting models. From what I can tell based on the tables, the approach relies on what appears to be a free ratio model, the use of which is generally discouraged due to it being overly parameter rich. A large number of likelihood ratio tests are then made between the free ratio model and models placing individual branches or clades in the ‘foreground’. This approach appears to (incorrectly) use the most parameter-rich model as the null model for the likelihood ratio tests. Models that restrict the ‘foreground’ branch/clade to ω = 1 are also used but they are also compared to the parameter rich model (rather than to same model but where the foreground ω is free to vary). As far as I can tell, no models are used that combine multiple branches or clades as might be expected. Beyond these issues, a much simpler approach could be used that also more directly tests the author’s hypotheses by comparing ω between mammals with and without pineal glands and between lineages with and without evidence for pseudogenization in that specific gene. A codon model specifically designed to detect relaxation of selective constraint (ie, RELAX) would be useful in this regard.
- It would be nice to see selection analyses on the crocodilian sequences as well to, for example, show that they are still under strong selective constraint. I think this could still be useful without other sequences for comparison (eg, from birds/turtles).
- I initially found the mix of analyses on mammalian and crocodilian genes unusual, but this made more sense within the context of animals with and without pineal glands. This could perhaps be made more clear, especially in the title which only mentions placental mammals and not crocodylians or the lack of pineal glands.
- I generally thought the figures were well put together quite effective, but the text size (most notably in Fig. 2) can be quite small and hard to read, even when zooming in.

Is the study design appropriate and does the work have academic merit?

Yes

Is the work clearly and accurately presented and does it cite the current literature?

Yes

If applicable, is the statistical analysis and its interpretation appropriate?

Partly

Are all the source data underlying the results available to ensure full reproducibility?

Partly

Are the conclusions drawn adequately supported by the results?

Yes

Are sufficient details of methods and analysis provided to allow replication by others?

Partly

Reviewer Expertise:

molecular evolution, comparative genomics, sensory biology

I confirm that I have read this submission and believe that I have an appropriate level of expertise to confirm that it is of an acceptable scientific standard, however I have significant reservations, as outlined above.

# Response

**Published:** 2021-11-24

**Authors:** Christopher Emerling

**Affiliations:** Reedley College, USA

**Author responses to Dr. Schott in bold text below with reviewer comments in italics.**

*In this study, the authors examined two melatonin synthesis genes (AANAT and ASMT) and two melatonin receptors (MTNR1A and MTNR1B) in 110 placental mammals and 13 crocodylians. They hypothesized that species which lack pineal glands, the primary source of melatonin production, would lack functional melatonin synthesis and receptor genes. The authors found support for this hypothesis in placental mammals, but not in crocodylians. In the 13 crocodylians examined, all of which are thought to lack pineal glands, the authors found intact melatonin genes and expression of two of those genes was confirmed with tissue-specific transcriptome sequencing in American alligator, specifically in the eye. In contrast, placental mammals thought to lack pineal glands (xenarthrans, pangolins, sirenians, and whales), as well as colugos and some fossorial mammal lineages, showed evidence for independent inactivation and loss of all four melatonin genes. Many other lineages also showed evidence of inactivation or loss in a smaller number of the genes. The authors speculate that loss of the melatonin synthesis and receptor genes may be associated with changes in thermoregulation and the loss of non-shivering thermogenesis. Overall, the manuscript is very well written, presents new and interesting results, and outlines several important areas for future research. I outline below several areas where the authors could further improve the paper:*

**We extend our thanks to Dr. Schott for spending the time to review our manuscript and giving generally encouraging feedback on our presentation. We appreciate his critical feedback and have done our best to appropriately address all of his comments below.**

*Inference of gene loss and pseudogenization depends on high quality sequencing data and genomic assemblies and apparent loss and pseudogenization could be due to incomplete sequencing/assembly or sequencing/assembly errors. While patterns of shared indels or premature stop codons are particularly convincing, I believe this caveat should still be addressed, especially considering that ~70% of the mammals examined were listed as having at least one pseudogenized or lost gene.*

**We agree that this can indeed be an issue with genomic data. While we are confident that the shared inactivating mutations, often paired with dN/dS estimates suggestive of relaxed selection, are strong evidence of pseudogenization in the pineal-less mammals and colugos, we believe Dr. Schott is correct that we should present additional evidence for the inactivation of genes in other taxa. To address this, we have done the following:**

1. **Collected additional genomic sequences from related taxa for species with seemingly unique inactivating mutations (i.e., to test for evidence of shared mutations).**
2. **In cases where this did not provide positive evidence of inactivating mutations (e.g., no adequate sister taxon, no shared mutations), we mapped short reads to the genomic contigs for such taxa.**

**We performed these analyses for all taxa that have evidence for inactivation of one or more melatonin synthesis gene and/or both melatonin receptor genes, given that these are the most relevant for our study. This is in contrast to the myriad examples of species that only have an *MTNR1A* or *MTNR1B* pseudogene, which would likely still have some, if not most, melatonin pathway functionality We have included these additional data in the Underlying Data and Extended Data in the form of Supplementary Table S13 and Supplementary Figures S8–S14.** **We believe our analyses effectively corroborate the inactivation of nearly every example of melatonin synthesis and/or signalling disruption, with the sole exception being for the pair of pseudogenic melatonin receptor genes in the Hawaiian monk seal *Neomonachus schauinslandi* (notably** **an unexpected finding).**

*Further to the point above, the sources of the genomic data used in the study is unclear. The main supplementary table of the genome sources does not provide much meaningful information (e.g., source listed as WGS). Looking through some of the subsequent SI tables accession numbers are given for some of the genes, but others are simply listed as New. Ideally all of the genomes would be made available, but minimally more detail needs to be provided on the genomic data (quality, completeness, etc…) used to obtain the sequences analyzed in the current study. It is difficult to evaluate the robustness of the results or reproduce the study without this.*

**We agree that this would be of utility to some readers. To address this, we have added more information to Supplementary Table S1, including the identifying prefix for assemblies found in NCBI, coverage, contig N50 and scaffold N50. For sequences assembled via short read mapping, we have included coverage data for each exon that we were able to assemble from short reads. Furthermore, since our novel sequences were derived from different genomic projects in different labs that have yet to publish their assemblies, we have indicated in the Supplementary Table S1 legend whom to contact (with e-mail addresses) for each relevant sequencing project.**

*It was also unclear whether the transcriptome data were newly sequenced or the analysis was of previously published data. More detail should be provided.*

**The transcriptome data for *A. mississippiensis *were** **derived from NCBI’s sequence read archive (SRA). Though this was described in the Underlying Data, we now have further clarified this in the revised manuscript in the following ways:**

- **A statement in the Materials and Methods on the generation of crocodylian sequences (on pg. 7).**
- **An explicit statement that the RNA sequencing data for the Alligator has been previously published with a reference to the corresponding paper (pg. 10).**

*The explanation of the methodology and approach used in the selection analyses was difficult to follow and perhaps overly complex. The authors mention a nested approach but do not provide much detail. Looking through the supplementary tables of PAML results I do not see nested sets of models or an attempt to find the best fitting models. From what I can tell based on the tables, the approach relies on what appears to be a free ratio model, the use of which is generally discouraged due to it being overly parameter rich. A large number of likelihood ratio tests are then made between the free ratio model and models placing individual branches or clades in the ‘foreground’. This approach appears to (incorrectly) use the most parameter-rich model as the null model for the likelihood ratio tests. Models that restrict the ‘foreground’ branch/clade to ω = 1 are also used but they are also compared to the parameter rich model (rather than to same model but where the foreground ω is free to vary). As far as I can tell, no models are used that combine multiple branches or clades as might be expected. Beyond these issues, a much simpler approach could be used that also more directly tests the author’s hypotheses by comparing ω between mammals with and without pineal glands and between lineages with and without evidence for pseudogenization in that specific gene. A codon model specifically designed to detect relaxation of selective constraint (ie, RELAX) would be useful in this regard.*

**We thank Dr. Schott for this critical feedback, as Dr. Janiak had the same concerns; it clearly needed to be clarified further. In short, we did not use a free ratio model as the null model. We designated a model in which certain branches were grouped based on their association with distinct inferences of pineal gland loss / gene losses, with branches being defined as functional (no evidence of dysfunction), pseudogenic (post-dating branch in which pseudogenization is inferred to have occurred), mixed/transitional (branch in which pseudogenization is inferred to have occurred), and pre-mutation (no evidence of pseudogenization on the branch but external evidence suggests gene may be under relaxed selection). As stated in the manuscript, this is following the approach of Meredith *et al.* 2009. Accordingly, there was a ‘master model’ for each gene, with each branch category described above, and this model was then compared to null models in which a specific pseudogene branch (or set of branches), mixed/transitional branch or pre-mutation branch was set to the background or 1, effectively testing whether a specific branch or set of branches was distinct from the background and/or 1, and therefore displaying evidence of relaxed selection. This was to test for individual historical cases of relaxed selection, rather than a broad ‘average’ pattern of relaxed selection, the latter of which does not accomplish what we were trying to test**. **Details of what we added can be found in the sections described below:**

1. **We have elaborated on the textual specifics of these methods under a section entitled “Evolutionary analyses” within the Materials and Methods (pgs. 9–10).**
2. **We have included four supplementary figures showing how each of the branch categories within the dN/dS analyses were designated within the ‘master’ model for each gene (Supplementary Figures S1–S4). We then paired the branch labeling with the dN/dS results in Supplementary Tables S7–S10 by use of numbering, to make for a smoother comparison of the two. **

**We believe that this should be far easier to understand than in our previous iteration, and hope it will satisfy Drs. Schott and Janiak.**

*It would be nice to see selection analyses on the crocodilian sequences as well to, for example, show that they are still under strong selective constraint. I think this could still be useful without other sequences for comparison (eg, from birds/turtles).*

**We agree with this suggestion and have done so. We added sections in the Materials and Methods (pgs 8 & 10) and Results (pg 13), added the alignments and topology to Supplementary Datasets S5–S9, and modelling results in Supplementary Tables S6 and S11. The basic result is that all four genes appear to be under purifying selection in crocodylians, with three ratio models (background, stem Crocodylia, crown Crocodylia) being statistically indistinguishable from one ratio models, and the crown and stem Crocodylia w estimates being very similar to the background w.**

*I initially found the mix of analyses on mammalian and crocodilian genes unusual, but this made more sense within the context of animals with and without pineal glands. This could perhaps be made more clear, especially in the title which only mentions placental mammals and not crocodylians or the lack of pineal glands.*

**Regarding the title, given that the first version of the manuscript is technically published via the Open Research Europe model, we thought changing it would make it confusing for future referencing purposes, especially as the manuscript has already been cited. Furthermore, given that the crocodylians we examined did not show evidence of inactivating melatonin pathway genes, including a crocodylian reference in the title seemed to us to take away from our most important finding, i.e., mentioning the absence of positive evidence for inactivation in crocs may detract from the title. However, we have tried to make the inclusion of crocodylians in our study clearer in the following ways:**

1. **Including an initial mention of the crocodylian results at the end of the introduction (pg 6).**
2. **Including a paragraph in the Materials and Methods describing the sequencing strategy for our novel crocodylian sequences (pg 8).**
3. **As discussed above, there are now paragraphs discussing the dN/dS analyses unique to crocs and other sauropsids in the Materials and Methods (pgs 8 & 10).**
4. **Including a section devoted to crocs in the Results (pg 13).**

*I generally thought the figures were well put together quite effective, but the text size (most notably in Fig. 2) can be quite small and hard to read, even when zooming in.*

**We are happy to hear that Dr. Schott found the figures to be effective. We have fixed the issues with text size as follows**

1. **We made the letter labels in Figure 1 larger and superimposed colored circles upon to make them stand out among the branches.**
2. **For Figure 2, we made species names larger and increased the resolution for the alignments and species names.**

# Peer review: Reviewer response for version 1

**DOI:** 10.21956/openreseurope.14874.r27204

**Published:** 2021-07-16

**Authors:** Mareike C. Janiak

**Affiliations:** 1 School of Science, Engineering and Environment, University of Salford, Salford, UK

In the manuscript “Genomic evidence for the parallel regression of melatonin synthesis and signaling pathways in placental mammals” the authors examine four genes related to melatonin synthesis and signaling in a broad sample of placental mammals, as well as 13 crocodylians. The authors pay special attention to species that (appear to) lack a pineal gland, the organ that secretes melatonin. Given the importance of melatonin in regulating an organism’s circadian rhythm, among other function, the authors hypothesized that the four genes involved in melatonin synthesis and signaling may remain functional even in species that lack a pineal gland, as there may be other sources of melatonin within the body.

 The authors show that, despite lacking a pineal gland, crocodylians show no disruptions to their melatonin synthesis and signaling genes and may synthesize melatonin within the eye. Several groups of placental mammals, however, appear to have disrupted melatonin synthesis and/or signaling pathways, including those lacking a pineal gland, living underground, and colugos.

 The authors discuss the implications of these findings and suggest directions for future research, such as potential links with temperature and thermoregulation. However, the authors also rightly point out that these results are surprising, considering the (assumed) importance of melatonin signaling and suggest that there may be other mechanisms for melatonin detection, or other pathways for circadian signaling.

 Overall, I found the manuscript to be very well-written and clearly presented, making it quite enjoyable to read. The figures are also very effective in communicating the results visually.

 My main suggestions for improving the manuscript are as follows:

1. The authors may be interested in the program RELAX (Wertheim et al., 2015; https://doi.org/10.1093/molbev/msu400) to explicitly test for relaxed selection on the pineal gland-less lineages. It seems like a useful test for the present dataset.
2. I found the description of the PAML analyses confusing and I’m not sure that I would be able to reproduce them based on the methods, specifically the paragraph describing the nested models and models for relaxed selection (note to the editor: it would be very useful if the manuscripts provided to reviewers included line numbers!). Also, it sounds like these included a large number of comparisons, were these results corrected for multiple comparisons? And if so, how?
3. The authors refer to all genes that show premature stop codons as “pseudogenes” even though this may not be what they are. There is growing evidence that so-called “pseudogenes” may in fact be functional, for example see Goodhead & Darby, 2015; https://doi.org/10.1016/j.mib.2014.11.012 and Cheetham et al., 2020; https://doi.org/10.1038/s41576-019-0196-1. While the authors are probably correct that these pseudogenes may no longer produce an equivalent product, I think it’s worth acknowledging the functional potential of pseudogenes.

Some minor suggestions:

1. I am a bit confused about the source of the crocodylian sequences, especially the transcriptomes. Based on the methods, it sounds as if these were sequenced by other researchers and retrieved from NCBI by the authors. However, the acknowledgements include thanks to a zoological park for crocodylian tissue samples. Are any of the results based on de novo sequencing? If so, it would be appropriate to describe this in detail in the methods.
2. The letters along the branches in Figure 1 are quite small and hard to read without zooming in. Could these be a bit bigger?
3. Finally, I am admittedly not an expert in melatonin pathway or circadian rhythm research, so I hope that others can provide more insightful feedback on the authors' discussion of the implications of their findings. In my non-expert opinion the authors appear appropriately cautious in how they interpret their findings, posing more questions than answers. 

Is the study design appropriate and does the work have academic merit?

Yes

Is the work clearly and accurately presented and does it cite the current literature?

Yes

If applicable, is the statistical analysis and its interpretation appropriate?

Yes

Are all the source data underlying the results available to ensure full reproducibility?

Yes

Are the conclusions drawn adequately supported by the results?

Yes

Are sufficient details of methods and analysis provided to allow replication by others?

Partly

Reviewer Expertise:

comparative genetics/genomics, dietary adaptations

I confirm that I have read this submission and believe that I have an appropriate level of expertise to confirm that it is of an acceptable scientific standard, however I have significant reservations, as outlined above.

## References

[1] : RELAX: detecting relaxed selection in a phylogenetic framework. *Mol Biol Evol* .2015;32(3) : 10.1093/molbev/msu400 820-32 10.1093/molbev/msu400 25540451PMC4327161

[2] : Taking the pseudo out of pseudogenes. *Curr Opin Microbiol* .2015;23: 10.1016/j.mib.2014.11.012 102-9 10.1016/j.mib.2014.11.012 25461580

[3] : Overcoming challenges and dogmas to understand the functions of pseudogenes. *Nat Rev Genet* .21(3) : 10.1038/s41576-019-0196-1 191-201 10.1038/s41576-019-0196-1 31848477

# Response

**Published:** 2021-11-24

**Authors:** Christopher Emerling

**Affiliations:** Reedley College, USA

**AUTHOR RESPONSES TO DR. JANIAK IN BOLD TEXT BELOW** **Author responses to Dr. Janiak in bold text below with reviewer comments in italics.**

*In the manuscript “Genomic evidence for the parallel regression of melatonin synthesis and signaling pathways in placental mammals” the authors examine four genes related to melatonin synthesis and signaling in a broad sample of placental mammals, as well as 13 crocodylians. The authors pay special attention to species that (appear to) lack a pineal gland, the organ that secretes melatonin. Given the importance of melatonin in regulating an organism’s circadian rhythm, among other function, the authors hypothesized that the four genes involved in melatonin synthesis and signaling may remain functional even in species that lack a pineal gland, as there may be other sources of melatonin within the body.*

*The authors show that, despite lacking a pineal gland, crocodylians show no disruptions to their melatonin synthesis and signaling genes and may synthesize melatonin within the eye. Several groups of placental mammals, however, appear to have disrupted melatonin synthesis and/or signaling pathways, including those lacking a pineal gland, living underground, and colugos.*

*The authors discuss the implications of these findings and suggest directions for future research, such as potential links with temperature and thermoregulation. However, the authors also rightly point out that these results are surprising, considering the (assumed) importance of melatonin signaling and suggest that there may be other mechanisms for melatonin detection, or other pathways for circadian signaling.*

*Overall, I found the manuscript to be very well-written and clearly presented, making it quite enjoyable to read. The figures are also very effective in communicating the results visually*.

**We thank Dr. Janiak for taking the time to review our manuscript and giving positive feedback on its overall presentation. We have done our best to adequately respond to her insightful comments below.**

*My main suggestions for improving the manuscript are as follows:*

1. *The authors may be interested in the program RELAX (Wertheim et al., 2015; https://doi.org/10.1093/molbev/msu400) to explicitly test for relaxed selection on the pineal gland-less lineages. It seems like a useful test for the present dataset.*

**We are indeed familiar with RELAX and have found it to be useful in certain analyses. However, we believe that PAML is sufficient in the context of this particular study in providing additional evidence of pseudogenization besides disrupting mutations. Detailed comparisons of PAML and RELAX within the context of pseudogene evolution to better understand how the analyses compare in estimating dN/dS during transitions from functional to nonfunctional genes would indeed be interesting to conduct. However, we think that this is beyond the scope of this empirical melatonin-focused paper.**

****

*I found the description of the PAML analyses confusing and I’m not sure that I would be able to reproduce them based on the methods, specifically the paragraph describing the nested models and models for relaxed selection (note to the editor: it would be very useful if the manuscripts provided to reviewers included line numbers!). Also, it sounds like these included a large number of comparisons, were these results corrected for multiple comparisons? And if so, how*

**We agree with this fair criticism on the point of the methods. Given that Dr. Schott made the same point, clearly this needed to be fixed to ensure replicability. We have made a concerted effort to improve comprehension of the methods in the following ways:**

1. **We have elaborated on the textual specifics of these methods under a section entitled “Evolutionary analyses” within the Materials and Methods (pgs. 9–10).**
2. **We have included four supplementary figures showing how each of the branch categories within the dN/dS analyses were designated within the ‘master’ model for each gene (Supplementary Figures S1–S4). We then paired the branch labeling with the dN/dS results in Supplementary Tables S7–S10 by use of numbering, to make for a smoother comparison of the two. **

**Regarding multiple test comparisons, we have applied the Holm-Bonferroni method for multiple testing. This is now detailed in the Materials and Methods (pg. 10), Results (pgs. 14 & 15) and the adjusted p-values are now included in Supplementary Tables S7–S10.**

*The authors refer to all genes that show premature stop codons as “pseudogenes” even though this may not be what they are. There is growing evidence that so-called “pseudogenes” may in fact be functional, for example see Goodhead & Darby, 2015; https://doi.org/10.1016/j.mib.2014.11.012 and Cheetham et al., 2020; https://doi.org/10.1038/s41576-019-0196-1. While the authors are probably correct that these pseudogenes may no longer produce an equivalent product, I think it’s worth acknowledging the functional potential of pseudogenes.*

**Thank you for this suggestion. Given that Cheetham *et al.* 2020 is more appropriate for our paper, we have included a reference to this study in the discussion (pg. 15), reproduced below:** “ **Despite this, given that recent discoveries have shown that presenting as a pseudogene does not always indicate that all biological function is lost (Cheetham *et al*., 2020), there remains a possibility that these apparently dysfunctional genes are able to contribute to melatonin metabolism in some unknown fashion.”**

*I am a bit confused about the source of the crocodylian sequences, especially the transcriptomes. Based on the methods, it sounds as if these were sequenced by other researchers and retrieved from NCBI by the authors. However, the acknowledgements include thanks to a zoological park for crocodylian tissue samples. Are any of the results based on de novo sequencing? If so, it would be appropriate to describe this in detail in the methods.*

**Most of the crocodylian genomic sequences were generated by us, with the exception of four draft genome assemblies ( *Alligator mississippiensis, A. sinensis, Gavialis gangeticus, Crocodylus porosus*),** **hence the acknowledgement of the St. Augustine Alligator Farm Zoological Park for tissue samples.** **The transcriptome data for *A. mississippiensis *were** **derived from NCBI’s sequence read archive (SRA). Though this was described in the Underlying Data, we now have further clarified this in the revised manuscript in the following ways:**

1. **A statement in the Materials and Methods on the generation of crocodylian sequences (on pg. 7).**
2. **An explicit statement that the RNA sequencing data for the Alligator has been previously published with a reference to the corresponding paper (pg. 10).**

*The letters along the branches in Figure 1 are quite small and hard to read without zooming in. Could these be a bit bigger?*

**We have addressed this by making the letters larger and superimposed colored circles upon to make them stand out among the branches. ** 

*Finally, I am admittedly not an expert in melatonin pathway or circadian rhythm research, so I hope that others can provide more insightful feedback on the authors' discussion of the implications of their findings. In my non-expert opinion the authors appear appropriately cautious in how they interpret their findings, posing more questions than answers.*

**We agree that this would have been ideal. We requested reviews from three different melatonin experts, but unfortunately none were available. Due to time constraints, we have decided to revise the manuscript with the reviews provided so far and will look forward to commentary from future studies.**
